# ﻿Two new species of *Penicillium* (Eurotiales, Aspergillaceae) and the first record of *P. danzhouense* from mangrove sediment in Thailand, with notes on antibacterial activity

**DOI:** 10.3897/mycokeys.126.172211

**Published:** 2025-12-22

**Authors:** Vassana Supapongsri, Ananya Sahathippayakul, Wanchat Sirisarn, Mohit Chand, Jui-Yu Chou, Piyangkun Lueangjaroenkit

**Affiliations:** 1 Department of Microbiology, Faculty of Science, Kasetsart University, Bangkok 10900, Thailand; 2 Department of Microbiology, Faculty of Medicine, Kasetsart University, Bangkok, 10900, Thailand; 3 International Program for Master of Science in Materials and Biological Technology, and Science Education, National Changhua University of Education, Changhua 500, Taiwan; 4 Department of Biology, National Changhua University of Education, Changhua 500, Taiwan; 5 Biodiversity Center Kasetsart University (BDCKU), Bangkok 10900, Thailand

**Keywords:** *
Exilicaulis*, *
Lanata-Divaricata*, mangrove sediment, new species, *
Penicillium
*, phylogenetic analysis

## Abstract

Two novel species of *Penicillium*, comprising *P. chanthaburiense***sp. nov.** and *P. buranakarlianum***sp. nov.**, were isolated from mangrove sediment collected at the King Rama IX International Mangrove Botanical Garden in Chanthaburi Province, Thailand. Morphological characterization combined with multilocus phylogenetic analyses of the internal transcribed spacer (ITS), β-tubulin (*TUB*), calmodulin (*CaM*), and RNA polymerase II second largest subunit (RPB2) genes placed *P. chanthaburiense***sp. nov.** as a new member of section Exilicaulis, series *Erubescentia*, while *P. buranakarlianum***sp. nov.** was assigned to section Lanata-Divaricata, series *Janthinella*. In addition, this study reports the first record of *P. danzhouense* from mangrove sediment in Thailand. Diagnostic morphological and molecular features distinguishing these taxa from their closest phylogenetic relatives are presented. These findings enrich the taxonomic framework of *Penicillium* and contribute to the understanding of fungal biodiversity in mangrove ecosystems. Furthermore, *P. chanthaburiense***sp. nov.** exhibited antibacterial activity against several clinically relevant Gram-positive pathogens, including methicillin-resistant *Staphylococcus aureus* (MRSA), highlighting the potential of mangrove-derived *Penicillium* species in antimicrobial research.

## ﻿Introduction

The genus *Penicillium* was first established by Johann Heinrich Friedrich Link in 1809 based on the characteristic brush-like conidiophores that define this morphologically distinct group. It is currently classified within the family Aspergillaceae, order Eurotiales, class Eurotiomycetes, and phylum Ascomycota (Houbraken et al. 2020). Species of *Penicillium* are globally distributed and commonly found in soil, decaying vegetation, and air, as well as in various indoor and food environments. They play essential ecological roles as decomposers and are also renowned for their industrial applications. For instance, *P. chrysogenum* is historically significant as the original source of the antibiotic penicillin (Gaynes 2017), while *P. camemberti* and *P. roqueforti* are indispensable in cheese ripening and flavor development (Ropars et al. 2020; Cleere et al. 2024). More recently, several *Penicillium* species have attracted attention as sources of novel bioactive compounds, industrial enzymes, and fermentation agents, highlighting their expanding biotechnological potential (Ifunanya et al. 2023; Shaaban et al. 2023; Lv and Zeng 2024).

Recent advancements in fungal systematics, particularly the use of multilocus phylogenetic analyses combined with detailed morphological observations, have refined the taxonomy of this large genus. Houbraken et al. (2020) proposed a comprehensive revision dividing *Penicillium* into two subgenera, *Penicillium* and *Aspergilloides*. This subgeneric structure is relatively uncommon among filamentous fungi and reflects deep evolutionary divergence within the genus. Each subgenus encompasses multiple sections that group species based on both phylogenetic relationships and shared morphological traits. The genus is further divided into 34 sections and 102 series (Thitla et al. 2025), providing a hierarchical framework that facilitates species identification, nomenclatural stability, and the recognition of cryptic diversity.

Among these, section Exilicaulis represents a taxonomically complex and morphologically variable group. The section was first formally established by Pitt (1980), with *P. restrictum* designated as the type species. It was originally defined to include species characterized by monoverticillate conidiophores and non-vesiculate stipes, traits that differentiated them from species with more elaborate or vesiculate structures. However, subsequent molecular studies have significantly broadened this definition. Recent multilocus phylogenetic analyses have demonstrated that species with biverticillate conidiophores, as well as those possessing solitary phialides, also belong within the phylogenetic boundaries of section Exilicaulis. As currently circumscribed, this section comprises six well-supported series: *Lapidosa*, *Corylophila*, *Restricta*, *Citreonigra*, *Alutacea*, and *Erubescentia*, reflecting the evolutionary diversity within the group (Thitla et al. 2025).

Section Lanata-Divaricata of the genus *Penicillium* was originally proposed by Thom (1930) and later reinstated by Houbraken and Samson (2011) based on phylogenetic evidence. This section is currently subdivided into five series: *Dalearum*, *Janthinella*, *Oxalica*, *Rolfsiorum*, and *Simplicissima* (Visagie et al. 2024). Species within this section are predominantly isolated from soil (Visagie et al. 2015; Diao et al. 2019), although they have also been recovered from various other substrates including air (Visagie et al. 2015), house dust (Visagie et al. 2014), pollen (Barbosa et al. 2022), and leaf litter (Houbraken et al. 2011). Members of section Lanata-Divaricata are typically characterized by divaricate to biverticillate conidiophores and colonies that tend to spread broadly on culture media (Nóbrega et al. 2024).

Mangrove ecosystems are recognised hotspots of fungal diversity, yet their microbial communities remain underexplored compared to terrestrial habitats. Investigating fungi from mangrove environments not only enriches our understanding of fungal taxonomy and ecology but also holds great promise for discovering novel bioactive compounds. Such natural products may serve as valuable sources of new antibiotics, particularly against resistant bacterial pathogens, addressing urgent global health challenges. Therefore, the present study aims to isolate and identify fungi of the genus *Penicillium* from the King Rama IX International Mangrove Botanical Garden in Chanthaburi, Thailand. Mangrove sediments were collected and subjected to fungal isolation, leading to the discovery of two novel *Penicillium* species and a new record of *P. danzhouense* from mangrove sediments in Thailand. Based on morphological observations combined with multilocus phylogenetic analyses of ITS, *TUB*, *CaM*, and *RPB2* sequences, two new species, *P. chanthaburiense* sp. nov. and *P. buranakarlianum* sp. nov., are described. Notes on their antibacterial activity are also provided, highlighting the potential of mangrove-derived *Penicillium* species for future antimicrobial research.

## ﻿Materials and methods

### ﻿Fungal isolation

**Figure 1.**
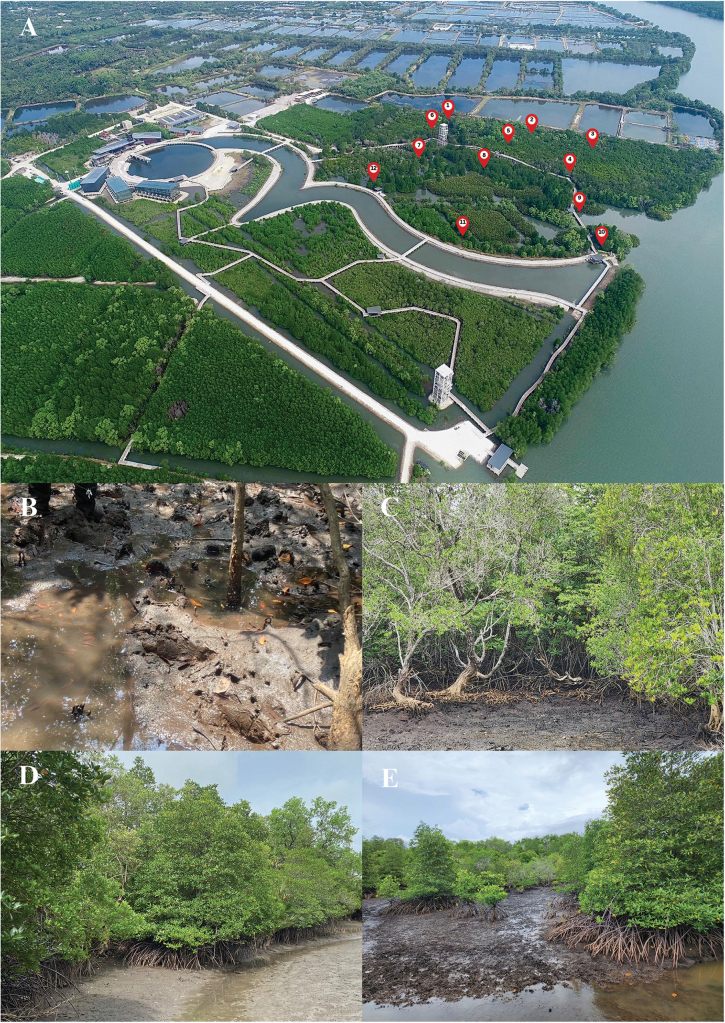
The King Rama IX International Mangrove Botanical Garden, Chanthaburi Province, Thailand. **A.** Map showing all 12 sampling sites; **B–E.** Representative sampling sites where the novel *Penicillium* species were frequently isolated; **B.** Sediment at sampling site 6; **C.** sampling site 7; **D.** sampling site 9; **E.** sampling site 12. Remarkably, both newly described *Penicillium* species were consistently recovered from sample sites 6 and 7. Photograph 1A courtesy of the Department of Marine and Coastal Resources (DMCR).

Mangrove sediments from the surface (0 cm) to a depth of 10 cm were collected from the King Rama IX International Mangrove Botanical Garden, Ban Samet Ngam, Nong Bua Subdistrict, Mueang District, Chanthaburi Province, Thailand (Fig. 1A) during the dry season (24–25 April 2023) and the rainy season (11–13 June 2023). A total of twelve sampling sites were designated using GPS coordinates (Fig. 1B–E and details shown in Table 1). The mangrove sediments were placed in sterile plastic bags, labelled with site information, and stored in an insulated box. All samples were transported to the laboratory under cool conditions (4–10 °C).

**Table 1.** Sampling locations of the twelve sites within the study area, including associated environmental parameters (temperature, pH, and salinity) recorded during both the dry and rainy seasons.

Sample sites	Latitude, Longitude	Temperature (°C)		pH		Salinity (PSU)	
		dry	rainy	dry	rainy	dry	rainy
1	12°31'43.9"N, 102°05'55.1"E	28.1	27.6	6.05	5.90	100	25
2	12°31'42.0"N, 102°05'57.6"E	29.0	27.8	5.32	5.58	85	15
3	12°31'39.9"N, 102°05'59.9"E	28.7	28.2	7.31	7.13	70	25
4	12°31'37.0"N, 102°05'57.5"E	31.7	28.4	5.69	5.87	115	30
5	12°31'40.0"N, 102°05'56.8"E	30.9	29.0	6.32	5.64	70	30
6	12°31'42.7"N, 102°05'54.1"E	30.5	28.3	5.57	5.44	80	35
7	12°31'40.8"N, 102°05'52.5"E	30.7	28.4	5.89	5.70	50	35
8	12°31'38.9"N, 102°05'55.5"E	33.0	28.5	5.54	5.40	105	40
9	12°31'35.6"N, 102°05'56.8"E	33.1	29.6	6.53	5.93	45	30
10	12°31'33.6"N, 102°05'55.6"E	33.7	31.1	4.89	5.83	40	15
11	12°31'37.4"N, 102°05'50.3"E	35.9	30.8	6.22	5.88	50	25
12	12°31'39.5"N, 102°05'49.1"E	35.0	32.9	6.00	5.19	60	35

Mangrove sediment samples were air-dried under shade and subjected to serial dilution plating, with dilution levels ranging from 1:100 to 1:1,000 in sterile distilled water. From each dilution, 100 µL aliquots were spread onto Potato Dextrose Agar (PDA) and Martin’s Rose Bengal Agar (MRBA). Both media were supplemented with streptomycin (100 µg/mL) to inhibit bacterial growth. The salinity and pH of the media were adjusted using sodium chloride (NaCl) to match the measured values of the corresponding sediment samples. Plates were incubated at room temperature (28–32 °C) for 3–5 days. Fungal colonies with distinct morphological characteristics were selected and purified using the hyphal tip isolation technique on PDA. The pure cultures were deposited and permanently preserved in a metabolically inactive state in two culture collection at the Thailand Bioresource Research Centre (TBRC) in Thailand and the NITE Biological Resource Centre (NBRC) in Japan.

### ﻿Morphological characterisation

Colony morphology, growth rate, sporulation, and pigment production were recorded after 7 days of incubation at 25 °C in darkness on various media, including malt extract agar (MEA), Czapek yeast extract agar with 20% sucrose (CY20S), Czapek yeast extract agar with 5% NaCl (CYAS), Czapek’s agar (CZ), oatmeal agar (OA), and creatine sucrose agar (CREA). In addition, growth on Czapek yeast extract agar (CYA) was also performed at 25 °C, 30 °C, and 37 °C in darkness for 7 days to determine the macroscopic colony characteristics. Colony colours (both obverse and reverse) were assessed using the Methuen handbook of colour (Kornerup and Wanscher 1967).

The microscopic features of the fungal isolates were assessed using a compound light microscope (Nikon Instech Co., Ltd., Tokyo, Japan). Specimens were stained with lactophenol cotton blue and observed for structural details. The characteristics of conidiophores, stipes, conidiogenous cells, conidia, and other relevant structures were examined. Digital images and morphometric data were captured and analysed using the NIS-Elements D imaging software (Nikon Instech Co., Ltd., Tokyo, Japan). To examine surface ultrastructure, scanning electron microscopy (SEM) was performed following the protocol outlined by Haranto et al. (2024). Fungi were cultured on potato dextrose agar (PDA) plates for 7–14 days, after which fungal colonies were cut into 1 × 1 cm sections. The samples were fixed overnight at 4 °C in 2% (v/v) glutaraldehyde prepared in 100 mM phosphate buffer (pH 7.2). After fixation, the samples were rinsed three times with the same buffer and then dehydrated through a graded ethanol series (10% to 100%) at one-hour intervals. The dehydrated specimens were subjected to critical point drying, sputter-coated with gold, and examined using a Quanta 450 scanning electron microscope (FEI, USA).

### ﻿DNA sequencing and phylogenetic analysis

Fungal isolates were grown on PDA at room temperature (25 ± 2 °C) for 3–5 days. Fresh mycelia (~50 mg) were harvested and transferred into 1.5 mL microcentrifuge tubes containing 700 µL of extraction buffer composed of 100 mM Tris-HCl (pH 8.0), 20 mM EDTA (pH 8.0), 1.4 M NaCl, 2% (w/v) cetyltrimethylammonium bromide (CTAB), and 4% (w/v) polyvinylpyrrolidone (PVP). The samples were homogenised using sterile micropestles and incubated at 65 °C for 30 minutes in a water bath. Following incubation, 700 µL of chloroform:isoamyl alcohol (24:1) was added and mixed thoroughly. The mixture was centrifuged at 12,000 rpm for 10 minutes, and the aqueous phase was transferred to a new tube. An equal volume of cold isopropanol was added to precipitate the DNA, followed by centrifugation at 12,000 rpm for 2 minutes. The resulting DNA pellet was washed with 300 µL of cold 70% ethanol, centrifuged again at 12,000 rpm for 2 minutes, and air-dried. The purified DNA was resuspended in 30 µL of deionised water and stored at −20 °C until use.

Genomic DNA extracted from fungal isolates was used as a template to amplify four genetic loci: the ITS region, *TUB*, *CaM*, *RPB2*. PCR amplification was performed using the following primer pairs: ITS1/ITS4 for ITS (White et al. 1990), Bt2a/Bt2b for *TUB* (Glass and Donaldson 1995), CMD5/CMD7 for *CaM* (Hong et al. 2006), and 5F/7CR for *RPB2* (Liu et al. 1999). The annealing temperatures for ITS, *TUB*, *CaM*, and *RPB2* were 52 °C, 52 °C, 58 °C, and 56 °C, respectively. Each PCR reaction was carried out in a final volume of 50 μL, containing 1× PCRBIO Taq Mix Red (PCR Biosystems, UK) and 0.5 μM of each primer. Thermal cycling conditions followed the protocol described by Leetanasaksakul et al. (2024).

PCR products were visualised on 1.5% agarose gels and subsequently purified using the MEGAquick-spin™ Plus Total Fragment DNA Purification Kit (iNtRON Biotechnology, Korea). Bidirectional sequencing was conducted by Bionics Inc. (Seoul, South Korea). Forward and reverse reads were assembled using MEGA version 11 (Tamura et al. 2021). The resulting consensus sequences were compared to existing sequences in the GenBank database using the BLASTn algorithm (Altschul et al. 1997) to determine similarity with known fungal taxa.

For phylogenetic analyses, ITS, *TUB*, *CaM*, and *RPB2* sequences of type strains and representative species of Penicillium (subgenus Aspergilloides) from sections *Exilicaulis* and *Lanata-Divaricata* were retrieved from GenBank (Suppl. material 1). Phylogenetic trees were reconstructed separately for each section based on concatenated alignments of the four loci (ITS, *TUB*, *CaM*, and *RPB2*) using the maximum likelihood (ML) method General Time Reversible (GTR) model implemented in MEGA11 (Tamura et al. 2021). Clade support was assessed by bootstrap analysis with 1,000 replicates (Felsenstein 1985). *P. anatolicum* (section Citrina) and *P. glabrum* (section Aspergilloides) were used as outgroup taxa for the trees of sections *Exilicaulis* and *Lanata-Divaricata*, respectively.

### ﻿Crude extract preparation

The three *Penicillium* species were each cultivated in 100 mL of Potato Dextrose Broth (PDB) in six individual Erlenmeyer flasks and incubated for one month at room temperature under shaking conditions at 200 rpm. Each flask was inoculated with approximately 10^5^ spores/mL of the respective fungal isolate. Following incubation, the cultures were filtered through Whatman No.1 filter paper to separate the mycelial biomass from the culture supernatant. The supernatant was extracted three times with an equal volume of ethyl acetate (1:1, v/v per extraction) (Díaz-González et al. 2025). The pooled ethyl acetate fractions were concentrated to dryness using a rotary evaporator at 40 °C. The remaining aqueous phase was freeze-dried to obtain the water-soluble fraction. Both ethyl acetate and aqueous extracts were stored and subsequently used for antibacterial activity assays.

### ﻿Antibacterial activity assay by agar well diffusion

The antibacterial activity of the fungal extracts was evaluated using the agar well diffusion method (Mousa et al. 2024). The crude extracts obtained from the ethyl acetate fraction were dissolved in 20% dimethyl sulfoxide (DMSO), while the aqueous extracts were dissolved in sterile distilled water. Both types of extracts were prepared at a final concentration of 10 mg/mL. A total of 13 bacterial strains, including standard strains and clinical isolates, were used for the assay: *Staphylococcus aureus* ATCC 25923, *S. aureus* ATCC 29213, methicillin-resistant *S. aureus* (clinical isolate), *Bacillus subtilis* ATCC 6051, *B. subtilis* 7988 (clinical isolate), *Bacillus cereus* ATCC 11778, *Escherichia coli* ATCC 25922, *E. coli* O157:H7 (clinical isolate), *Pseudomonas aeruginosa* ATCC 27853, *Klebsiella pneumoniae* ATCC 70063, *Shigella enterotidis* (clinical isolate), *Salmonella enterica* serotype Typhi (clinical isolate), and *Vibrio cholerae* (clinical isolate). Each bacterial suspension was adjusted to a turbidity equivalent to 0.5 McFarland standard (1.5 × 10^8^ CFU/mL) and uniformly spread onto the surface of Mueller-Hinton agar plates using a sterile cotton swab. Wells with diameter of 6 mm were aseptically punched into the agar, and 100 µL of each extract solution was carefully loaded into the wells. Streptomycin at a concentration of 10 mg/mL was used as a positive control, while 20% DMSO served as a negative control. The plates were then incubated at 37 °C for 18 to 24 hours. The antibacterial activity was assessed by measuring the diameter of the clear inhibition zones around each well in millimetres. All experiments were conducted in triplicate to ensure reproducibility.

## ﻿Results

A total of 160 fungal isolates were recovered from mangrove sediments at the King Rama IX International Mangrove Botanical Garden, Chanthaburi, Thailand. Among these, *Penicillium* was the dominant genus, comprising 68 isolates (42.5%). Sixteen isolates were preliminarily identified as *Penicillium* based on morphology and were of particular interest because they displayed distinct morphological traits that differed from known species in the genus. Sequences of ITS, *TUB*, *CaM*, and *RPB2*, combined with multilocus phylogenetic analyses, provided a framework to assess their taxonomy and supported the recognition of new species within sections *Exilicaulis* and *Lanata-Divaricata*.

### ﻿Novel species delineation and identification

**Figure 2.**
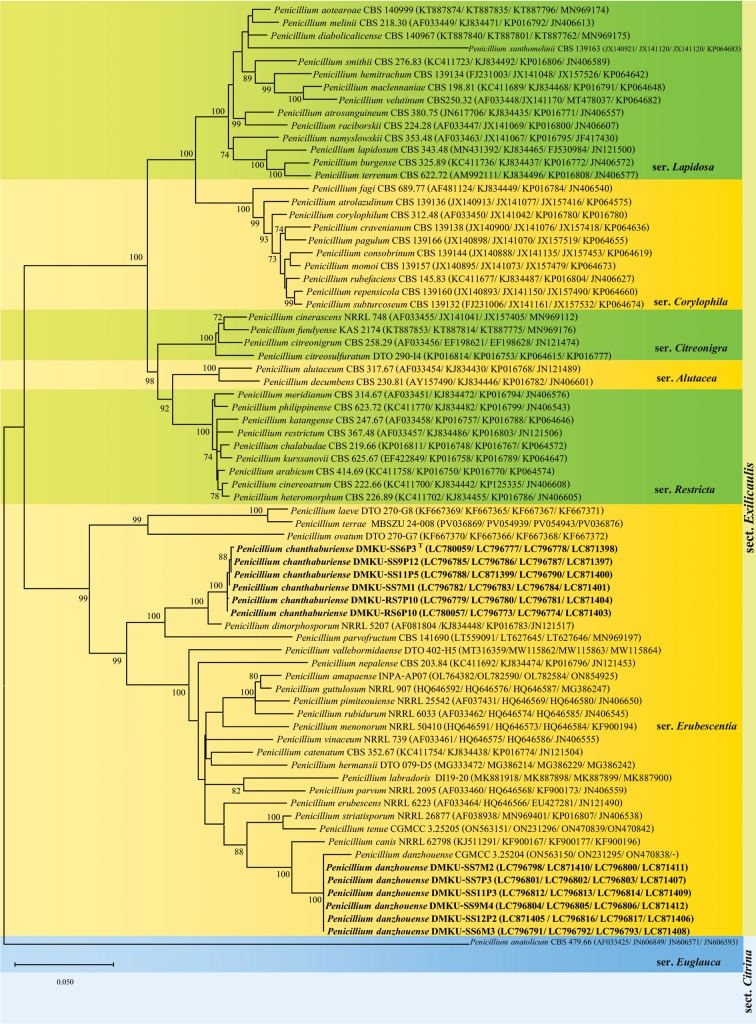
Maximum likelihood (ML) phylogenetic tree of Penicillium species in section Exilicaulis, based on a concatenated dataset of ITS, *TUB*, *CaM*, and *RPB2* sequences. The tree illustrates the phylogenetic positions of the newly described species relative to closely related taxa. Bootstrap support values ≥ 70% are indicated at the nodes. Strain numbers follow species names, with GenBank accession numbers for ITS, *TUB*, *CaM*, and *RPB2* provided in parentheses. *Penicillium anatolicum* CBS 479.66 was used as the outgroup. Scale bar: patristic distance of 0.050.

Multilocus sequence comparisons revealed distinct patterns among the 16 isolates. Six fungal isolates—two from the rainy season (DMKU-RS6P10, DMKU-RS7P10) and four from the dry season (DMKU-SS6P3^T^, DMKU-SS7M1, DMKU-SS9P12, DMKU-SS11P5)—showed ITS sequences that were identical or differed by only two nucleotides. *TUB* and *RPB2* sequences were identical or varied by a single nucleotide, while *CaM* sequences were completely identical. Compared with *P. dimorphosporum* NRRL 52071, ITS and *TUB* sequences shared 97.9% similarity (11 and 9 nucleotide differences, respectively), *CaM* sequences showed 96.0% similarity (28 differences), and *RPB2* sequences were 99.0% similar (9 differences). Phylogenetic analysis was conducted on 73 Penicillium taxa from section Exilicaulis, including the six isolates using a combined dataset of ITS, *TUB*, *CaM*, and *RPB2* sequences. Maximum likelihood analysis placed the six new isolates in a distinct phylogenetic position, separate from other recognised species, with strong bootstrap support (100%). These strains formed a sister clade with *P. dimorphosporum* NRRL 52071 and belonged to the series *Erubescentia*. (Fig. 2), supporting their recognition as a novel species.

Another six fungal isolates collected during the dry season (DMKU-SS6M3, DMKU-SS7M2, DMKU-SS7P3, DMKU-SS9M4, DMKU-SS11P3, and DMKU-SS12P2) showed identical sequences across all four loci (ITS, *TUB*, *CaM*, and *RPB2*). BLASTn searches indicated 100% similarity of ITS and *TUB* with *Penicillium danzhouense* CGMCC 3.25204, while *CaM* showed 99.8% similarity (1 nucleotide difference). Phylogenetic analysis of section Exilicaulis using a concatenated ITS, *TUB*, *CaM*, and *RPB2* dataset confirmed that all six isolates belong to *P. danzhouense* within the Series *Erubescentia* clade, representing the first report of this species from mangrove sediments in Thailand. Notably, while *P. danzhouense* was previously reported based on a single isolate from tidal flat sediments in China, the recovery of six independent isolates in the present study provides strong evidence for its establishment and adaptation in mangrove sediment ecosystems.

**Figure 3.**
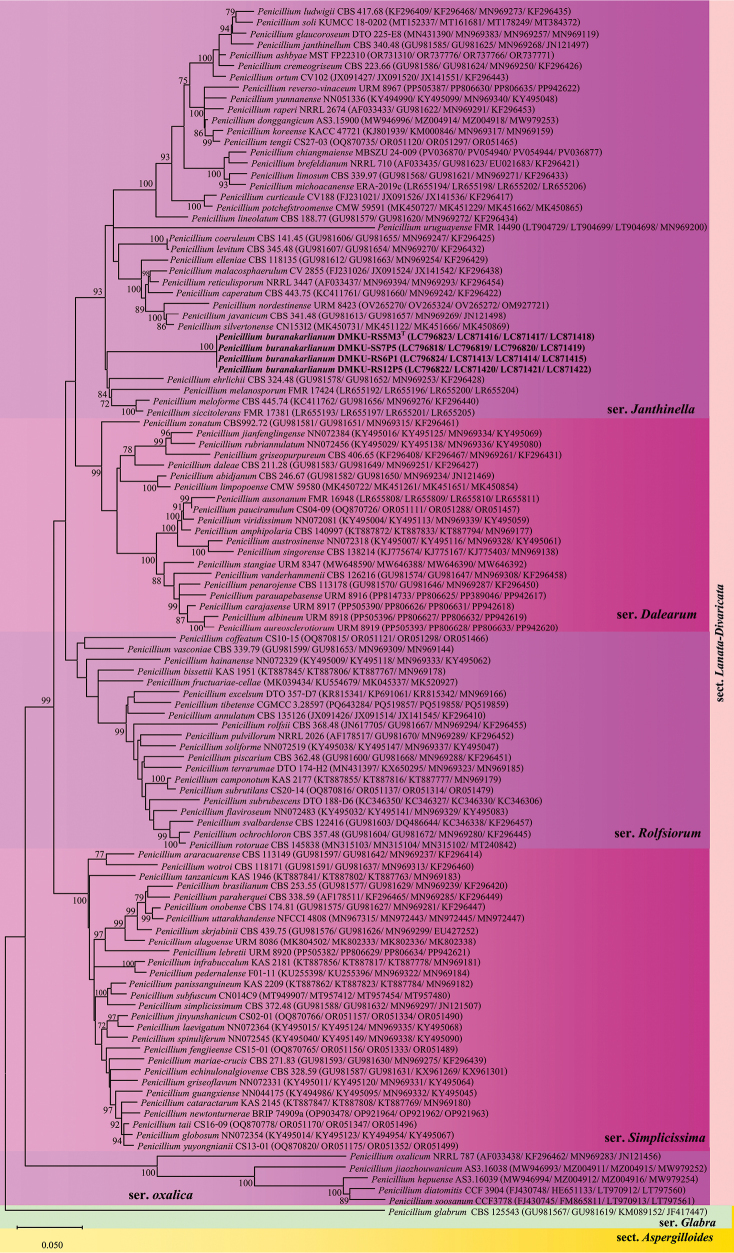
Maximum likelihood (ML) phylogenetic tree of Penicillium species in section Lanata-Divaricata, based on a concatenated dataset of ITS, *TUB*, *CaM*, and *RPB2* sequences. The tree illustrates the phylogenetic positions of the newly described species relative to closely related taxa. Bootstrap support values ≥ 70% are indicated at the nodes. Newly described species are shown in bold. Strain numbers follow species names, with GenBank accession numbers for ITS, *TUB*, *CaM*, and *RPB2* provided in parentheses. *Penicillium glabrum* CBS 125543 was used as the outgroup. Scale bar: patristic distance of 0.050.

Finally, four isolates—three fungal isolates collected during the rainy season (DMKU-RS5M3^T^, DMKU-RS6P1, and DMKU-RS12P5) and one from the dry season (DMKU-SS7P5). Phylogenies of series Janthinella within section Lanata-Divaricata were reconstructed from individual datasets of the ITS, *TUB*, *CaM*, and *RPB2* genes (Suppl. material 2), as well as from a concatenated dataset of these genes comprising 112 representative taxa across five recognized series (*Janthinella*, *Rolfsiorum*, *Dalearum*, *Simplicissima*, and *Oxalica*), with *P. glabrum* (section Aspergilloides, series *Glabra*) as the outgroup. In both the single-gene and combined analyses, the four isolates formed a strongly supported clade closely related to, but distinct from, *P. ehrlichii*, *P. melanosporum*, *P. meloforme*, and *P. siccitolerans* (Fig. 3). The combined phylogeny further indicated that these strains belong to series Janthinella (section Lanata-Divaricata) and supports their recognition as a novel species.

### ﻿Taxonomy

#### ﻿ Penicillium chanthaburiense

Taxon classificationFungiEurotialesAspergillaceae

Supapongsri, Sahathippayakul & Lueangjaroenkit sp. nov.

1C8F3732-4143-5EFC-9522-E363C57095DA

859666

##### Remark.

In: subgenus Aspergilloides, section Exilicaulis series *Erubescentia*.

##### Etymology.

The specific epithet “*chanthaburiense*” refers to the type locality, which is in Chanthaburi Province in Thailand.

##### Holotype.

Thailand • Chanthaburi Province, Mueang District, Nong Bua Subdistrict, Ban Samet Ngam, King Rama IX International Mangrove Botanical Garden; isolated from mangrove sediment during the dry season, 24 April 2023, V. Supapongsri, A. Sahathippayakul & P. Lueangjaroenkit; holotype, TBRC 18801, isotype NBRC 116997, ex-type living culture, DMKU-SS6P3, metabolically inactive culture preserved state, TBRC 18801 and NBRC 116997. GenBank accession numbers. ITS: LC780059; *TUB*: LC796777; *CaM*: LC796778; *RPB2*: LC871398.

##### Colony diam.

(in mm) 7 days, 25 °C: CYA 19-20, MEA 22-25, CY20S 18-20, CYAS 15-16, CZ 13-16, OA 21-23, CREA 12-13 and PDA 23-26. 7 days, 30 °C: CYA 19-21. 7 days, 37 °C: CYA 12-14, MEA 14 days 36-42, CZ 14 days 24-28.

##### Culture characteristics.

**Figure 4.**
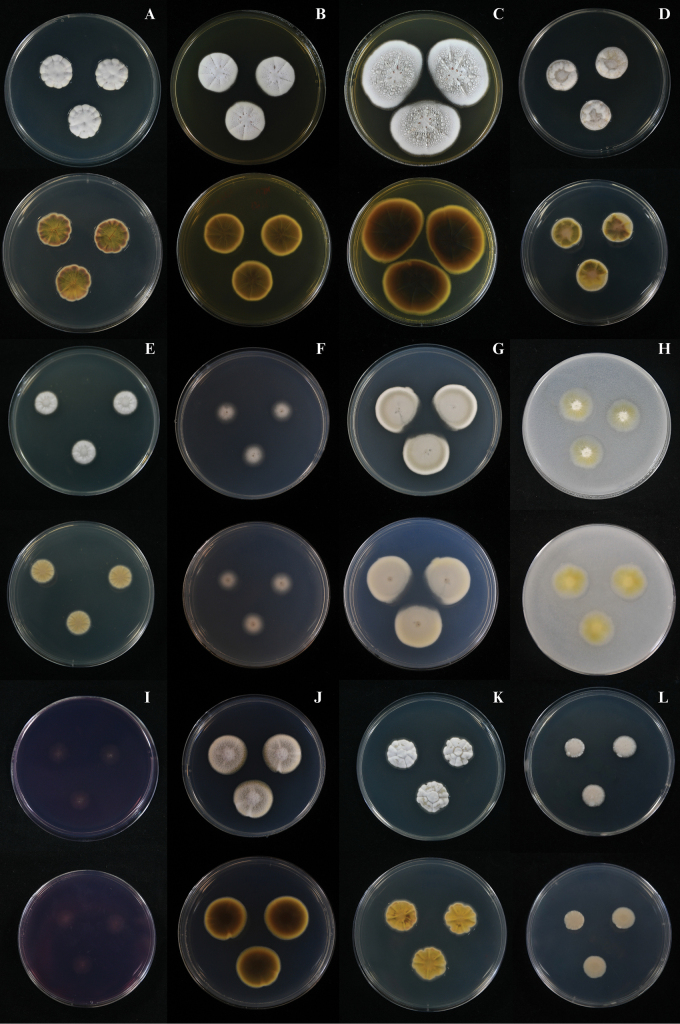
*Penicillium chanthaburiense* DMKU-SS6P3^T^. **A, B, D–F, H–J.** Colonies grown for 7 days at 25 °C on CYA, MEA, CY20S, CYAS, CZ, OA, CREA, and PDA, respectively; **C, G.** Colonies grown for 14 days at 25 °C on MEA and CZ, respectively; **K, L.** Colonies grown for 7 days on CYA at 30 °C and 37 °C, respectively. For each fungus, the images in the top row show the colony morphology from the front (surface view), while the images in the bottom row depict the reverse of the colony (underside view).

Colonies on CYA at 25 °C were slightly raised after 7 days, radially sulcate; margins undulate; mycelium white (1A1); texture velvety; sporulation very sparse, conidia *en masse* not determined; sclerotia and exudate absent; no soluble pigments produced; reverse coloration olive brown (4D8) at the centre, becoming dark brown (9F8) toward the margins (Fig. 4A). Colonies on MEA at 25 °C were slightly raised after 7 days, radially sulcate; margins undulate; mycelium greyish white (1B1); texture velvety; sporulation very sparse, conidia *en masse* not determined; sclerotia absent; exudate hyaline; no soluble pigments produced; reverse chocolate brown (6F4) at the centre, light yellow (4A5) toward the margins (Fig. 4B). Colonies on MEA at 25 °C were slightly raised after 14 days, radially sulcate; margins undulate; mycelium greyish white (1B1); texture velvety; sporulation moderately dense, conidia *en masse* light grey (1C1); sclerotia absent; exudate mostly hyaline, occasionally pale red (12A5); no soluble pigments produced; reverse yellowish brown (5F8) at the centre, light yellow (4A5) toward the margins (Fig. 4C). On CY20S after 7 days: colonies were slightly raised; margins undulate; mycelium greyish white (1B1); texture velvety to floccose; sporulation very sparse, conidia *en masse* not determined; sclerotia and exudate absent; no soluble pigments produced; reverse pale yellow (4A3) at the centre, olive brown (4E8) at the margins (Fig. 4D). On CYAS after 7 days: colonies were flat, slightly raised at the centre; margins undulate; mycelium white (1A1); texture velvety; sporulation very sparse, conidia *en masse* not determined; sclerotia and exudate absent; no soluble pigments produced; reverse champagne (4A4) (Fig. 4E). On CZ after 7 days: colonies were slightly raised; margins undulate; mycelium white (1A1); texture velvety; sporulation very sparse, conidia *en masse* not determined; sclerotia and exudate absent; no soluble pigments produced; reverse white (1A1) (Fig. 4F). On CZ after 14 days: colonies were slightly raised; margins undulate; mycelium white (1A1); texture velvety; sporulation moderately dense, conidia *en masse* grey (3B1) to olive grey (3D2); sclerotia and exudate absent; reverse grey (3B1) (Fig. 4G). On OA after 7 days: colonies were plane; margins undulate; mycelium white (1A1) at the centre, golden blonde (4B4) at the margins; texture felty; sporulation absent to very sparse, conidia *en masse* not determined; sclerotia and exudate absent; no soluble pigments produced; reverse chamois (4B5) (Fig. 4H). On CREA after 7 days: poor growth; acid production absent (Fig. 4I). On PDA after 7 days: colonies were umbonate; margins undulate; mycelium pale yellow (4A3); texture velvety; sporulation moderately dense, conidia *en masse* grey (2C1); sclerotia and exudate absent; no soluble pigments produced; reverse olive brown (4F8) at the centre, greyish yellow (3B4) at the margins (Fig. 4J). On CYA at 30 °C after 7 days: colonies were slightly umbonate, radially sulcate; margins undulate; mycelium white (1A1); texture velvety; sporulation very sparse, conidia *en masse* not determined sclerotia and exudate absent; no soluble pigments produced; reverse butter yellow (4A5) (Fig. 4K). On CYA at 37 °C after 7 days: colonies were slightly raised; margins filiform; mycelium yellowish white (3A2); texture floccose; sporulation very sparse, conidia *en masse* not determined sclerotia and exudate absent; no soluble pigments produced; reverse yellowish white (4A2) (Fig. 4L).

##### Micromorphology.

**Figure 5.**
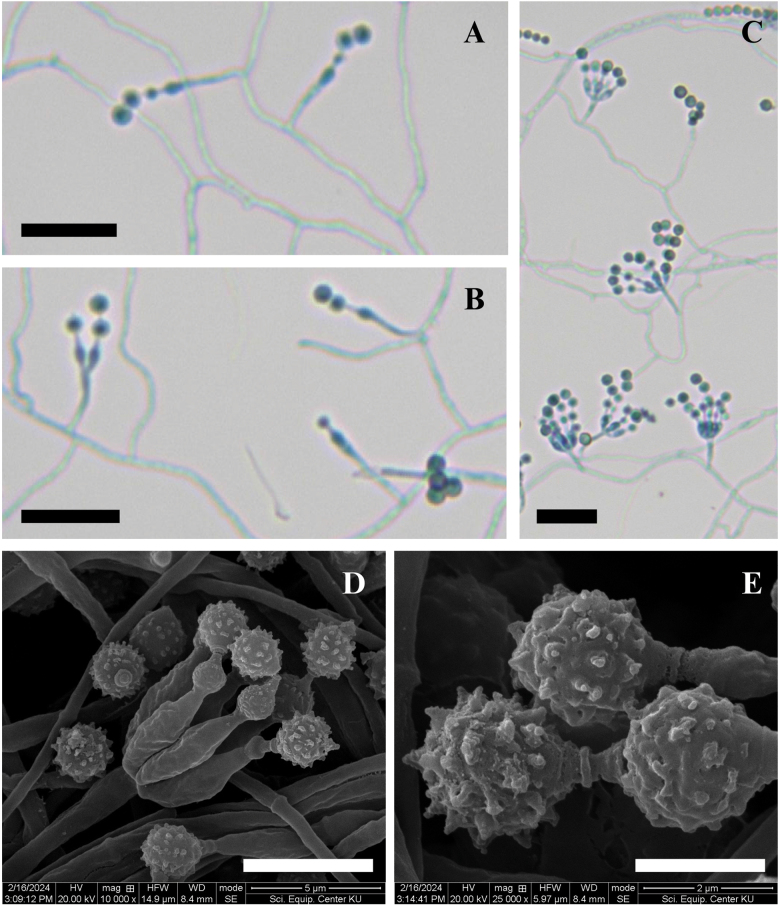
Morphology of *Penicillium chanthaburiense* DMKU-SS6P3^T^. **A–C.** Conidiophores are monoverticillate, unbranched, smooth, and hyaline. Phialides are ampulliform, arising singly or in groups; **D, E.** Conidia are globose with distinct spinose ornamentation visible under SEM. No sclerotia or sexual morph were observed. Scale bars: 15 µm (**A–C**); 5 µm (**D**); 2 µm (**E**).

Conidiophores are monoverticillate, unbranched, smooth-walled, and hyaline, measuring 6.5–15.0 × 1.0–2.0 μm. Phialides are ampulliform, arising singly or in groups of up to six on each conidiophore, smooth-walled and hyaline, measuring 4.0–7.0 × 1.5–2.5 μm (Fig. 5A–D). Conidia are globose measuring 2.0–3.0 μm in diameter. Under scanning electron microscopy (SEM), conidia appear globose and distinctly spinose (Fig. 5D, E). Sclerotia were not observed, and no sexual morph was present.

##### Habitat and distribution.

Mangrove sediment; only known from Chanthaburi Province, Thailand.

##### Notes.

*Penicillium chanthaburiense* is assigned to section Exilicaulis, series *Erubescentia*. In the phylogenetic tree (Fig. 2), *P. chanthaburiense* forms a well-supported sister clade to *P. dimorphosporum* (Swart, 1970). Despite their genetic proximity, the two species exhibit distinct morphological differences, particularly in colony characteristics. A key microscopic difference is the dimorphic conidia observed in *P. dimorphosporum*, while *P. chanthaburiense* consistently produced monomorphic conidia. On Czapek agar (CZ) after 14 days of incubation, *P. dimorphosporum* showed slower growth, reaching approximately 20 mm in diameter, whereas *P. chanthaburiense* attained 24–28 mm. The colony of *P. dimorphosporum* developed from white to pale greyish green, with a deep reddish-brown centre and white margin. In contrast, *P. chanthaburiense* displayed grey (3B1) to olive grey (3D2) mycelium at the centre with white (1A1) margins. Notably, *P. dimorphosporum* produced abundant pale pink exudate that intensified to deep red and secreted a deep reddish brown soluble pigment—features absent in *P. chanthaburiense*. The reverse colony colour also differed, being reddish brown in *P. dimorphosporum* and grey (3B1) in *P. chanthaburiense*. On malt extract agar (MEA), *P. dimorphosporum* exhibited colony development similar to that on CZ, including yellow-brown reverse coloration and exudate ranging from amber to pale pink. In contrast, *P. chanthaburiense* formed greyish white (1B1) mycelium, with hyaline to occasionally pale red (12A5) exudate, and a reverse colouration of centrally yellowish brown (5F8) surrounded by light yellow (4A5) at the margins. These morphological distinctions, together with phylogenetic evidence, support the recognition of *P. chanthaburiense* as a novel species within series *Erubescentia*. *P. chanthaburiense* showed the closest similarity to *P. dimorphosporum* NRRL 52071, differing by 11 nucleotide substitutions (2.1%) in ITS, 9 substitutions (2.1%) in *TUB*, 28 substitutions (4.0%) in *CaM*, and 9 substitutions (1.0%) in *RPB2*.

#### ﻿ Penicillium danzhouense

Taxon classificationFungiEurotialesAspergillaceae

C. Liu, Z.Q. Zeng & W.Y. Zhuang, 2023

E540C371-BC8A-59CE-9868-E16C9CCA779A

##### Remark.

In: subgenus Aspergilloides, section Exilicaulis series *Erubescentia*.

##### Colony diam.

(in mm) 7 days, 25 °C: CYA 18-19, MEA 20-23, CY20S 15-17, CYAS 8-10, CZ 15-16, OA 19-20, CREA 16-17 and PDA 18-19. 7 days, 30 °C: CYA 17-20. 7 days, 37 °C: CYA 10-12.

##### Culture characteristics.

**Figure 6.**
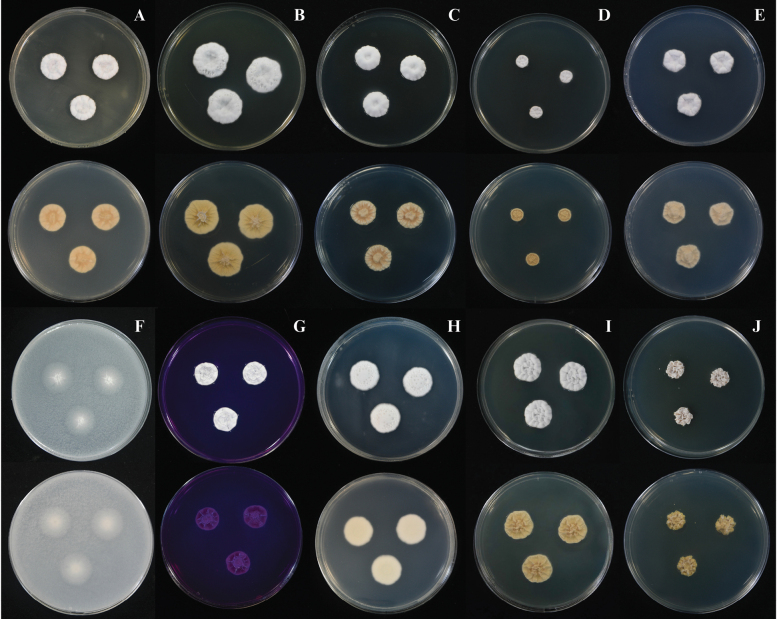
*Penicillium danzhouense* DMKU-SS7M2. **A–H.** Colonies grown for 7 days at 25 °C on CYA, MEA, CY20S, CYAS, CZ, OA, CREA, and PDA, respectively; **I, J.** Colonies grown for 7 days on CYA at 30 °C and 37 °C, respectively.

On CYA at 25 °C after 7 days, colonies were slightly raised with undulate margins; mycelium white (1A1), velvety in texture; sporulation very sparse, conidia *en masse* not determined sclerotia absent; exudate hyaline; no soluble pigments produced; reverse pale yellow (4A3) (Fig. 6A). On MEA after 7 days, colonies were crateriform with undulate margins; mycelium white (1A1), texture velvety; sporulation moderately dense, conidia *en masse* greyish white (2B1); sclerotia absent; exudate hyaline; no soluble pigments produced; reverse champagne (4A4) (Fig. 6B). On CY20S after 7 days, colonies were crateriform with undulate margins; mycelium white (1A1), velvety; sporulation very sparse, conidia *en masse* not determined; sclerotia absent; exudate hyaline; no soluble pigments produced; reverse champagne (4A4) (Fig. 6C). On CYAS after 7 days, colonies were slightly raised with undulate margins; mycelium white (1A1), velvety; sporulation very sparse, conidia *en masse* not determined; sclerotia and exudate absent; no soluble pigments produced; reverse pale yellow (4A3) (Fig. 6D). On CZ after 7 days, colonies were crateriform with undulate margins; mycelium white (1A1), velvety; sporulation very sparse, conidia *en masse* not determined; sclerotia and exudate absent; no soluble pigments produced; reverse yellowish white (4A2) (Fig. 6E). On OA after 7 days, colonies were plain with undulate margins; mycelium white (1A1), felty; sporulation absent to very sparse, conidia *en masse* not determined; sclerotia and exudate absent; no soluble pigments produced; reverse white (1A1) (Fig. 6F). On CREA after 7 days, colonies were slightly raised with undulate margins; mycelium white (1A1), velvety; sclerotia and exudate absent; no soluble pigments produced; acid production absent; base production present (Fig. 6G). On PDA after 7 days, colonies were slightly raised with undulate margins; mycelium white (1A1), velvety; sporulation moderately dense, conidia *en masse* greyish white (2B1); sclerotia absent; exudate hyaline; no soluble pigments produced; reverse yellowish white (2A2) (Fig. 6H). On CYA at 30 °C after 7 days, colonies were slightly raised, radially sulcate with undulate margins; mycelium white (1A1), velvety; sporulation very sparse, conidia *en masse* not determined; sclerotia and exudate absent; no soluble pigments produced; reverse pale yellow (4A3) (Fig. 6I). On CYA at 37 °C after 7 days, colonies were umbonate with undulate margins; mycelium white (1A1), velvety; sporulation absent to very sparse, conidia *en masse* not determined; sclerotia and exudate absent; no soluble pigments produced; reverse champagne (4A4) (Fig. 6J).

##### Micromorphology.

**Figure 7.**
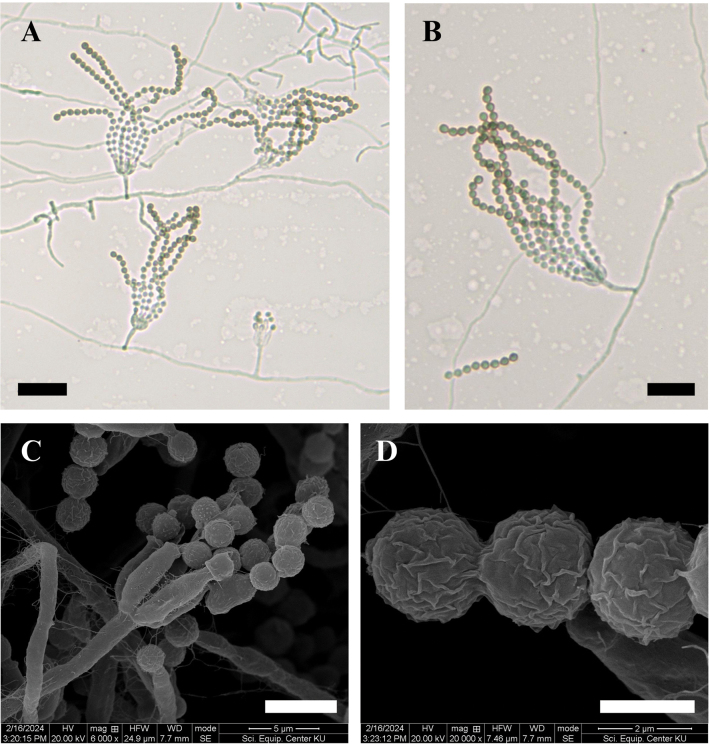
Morphological features of *Penicillium danzhouense* DMKU-SS7M2. **A, B.** Conidiophores are monoverticillate, unbranched, smooth-walled, and hyaline; **C.** Phialides are ampulliform, occurring singly or in groups of up to seven per conidiophore, smooth-walled, and hyaline; **D.** Conidia are globose with distinctly rugose surfaces as shown by SEM. Scale bars: 20 µm (**A**); 15 µm (**B**); 5 µm (**C**); 2 µm (**D**).

Conidiophores monoverticillate, unbranched, smooth-walled, hyaline, measuring 7.0–24.0 × 1.0–2.0 µm (Fig. 7A, B). Phialides ampulliform, occurring singly or in groups of up to seven per conidiophore, smooth-walled, hyaline, measuring 5.0–10.5 × 1.5–3.0 µm (Fig. 7C). Conidia globose measuring 2.0–3.0 µm. Under scanning electron microscopy (SEM), conidia appear globose and distinctly rugose (Fig. 7D). Sclerotia not observed. Sexual morph absent.

##### Habitat and distribution.

Tidal flat sediment in Hainan Province, China in 2020 and Mangrove sediment in Chanthaburi Province, Thailand in 2023.

##### Note.

*Penicillium danzhouense* was isolated from Thailand and represents the first record of this species in the country, and only the second report worldwide. Phylogenetic analysis based on a multilocus dataset placed our isolates within the same clade as *P. danzhouense* CGMCC 3.25204, the ex-type strain (Fig. 2), with strong statistical support. The morphological characteristics of our isolates closely correspond to the description of *P. danzhouense* by Liu et al. (2023), particularly in conidial size (2–3 μm), colony colours on CYA, MEA and PDA (white), reverse colony colours (light yellow), and colony diameter on PDA, all of which fall within the known range of the species. The environmental conditions of the sampling sites were also comparable, as the species was previously reported from tidal flat sediment in China and is here isolated from mangrove sediment in Thailand.

However, minor morphological deviations were observed when compared with the holotype. The conidiophores of our isolates tend to be shorter (7–24 μm vs. 12–40 μm in the holotype), whereas the phialides are slightly larger (5–10.5 μm vs. 4.6–8.7 μm). The number of phialides per conidiophore also varied slightly (1–7 in our isolates vs. 2–5 in the holotype). Growth characteristics on different media additionally showed subtle differences: colonies on CYA grew more slowly than the holotype (18–19 mm vs. 21–24 mm), whereas growth on MEA (20–23 mm vs. 14–17 mm) and on CYA at 37 °C (10–12 mm vs. 6–7 mm) was comparatively faster. Overall, the congruence of phylogenetic evidence and morphological similarity, with only minor intraspecific variations, supports the identification of these isolates as *Penicillium danzhouense*.

#### ﻿ Penicillium buranakarlianum

Taxon classificationFungiEurotialesAspergillaceae

Supapongsri, Sahathippayakul & Lueangjaroenkit sp. nov.

4ECF44E3-A4DF-5783-BEB7-FDD32BDA0971

859669

##### Remark.

In: subgenus Aspergilloides, section Lanata-Divaricata series *Buranakarliana*.

##### Etymology.

The specific epithet “buranakarlianum” is named in honour of Associate Professor Dr. Lerluck (Buranakarl) Chitadon, a senior faculty member of the Department of Microbiology, Faculty of Science, Kasetsart University. She served as the academic advisor for Assistant Professor Dr. Piyangkun Lueangjaroenkit from his undergraduate through to doctoral studies. Her guidance, knowledge, and unwavering support profoundly shaped his academic journey and she continues to encourage and inspire the authors.

##### Holotype.

Thailand • Chanthaburi Province, Mueang District, Nong Bua Sub-district, Ban Samet Ngam, King Rama IX International Mangrove Botanical Garden; isolated from mangrove sediment during the rainy season, 11 June 2023, V. Supapongsri, A. Sahathippayakul & P. Lueangjaroenkit; holotype, TBRC 18803, isotype NBRC 116999, ex-type living culture, DMKU-RS5M3, metabolically inactive culture preserved state, TBRC 18803 and NBRC 116999. GenBank accession numbers. ITS: LC796823; *TUB*: LC871416; *CaM*: LC871417; *RPB2*: LC871418.

##### Colony diam.

(in mm) 7 days, 25 °C: CYA 40-46, MEA 48-54, CY20S 41-46, CYAS 16-18, CZ 23-26, OA 43-47, CREA 33-35 and PDA 40-50. 7 days, 30 °C: CYA 40-46. 7 days, 37 °C: CYA no growth.

##### Culture characteristics.

**Figure 8.**
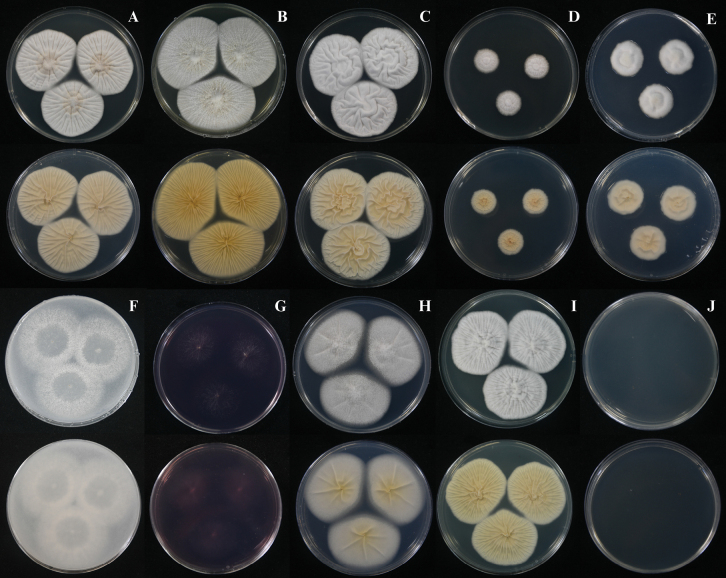
*Penicillium buranakarlianum* DMKU-RS5M3^T^. **A–H.** Colonies grown for 7 days at 25 °C on CYA, MEA, CY20S, CYAS, CZ, OA, CREA, and PDA, respectively; **I.** Colonies grown for 7 days on CYA at 30 °C; **J.** No growth on CYA at 37 °C.

**Figure 9.**
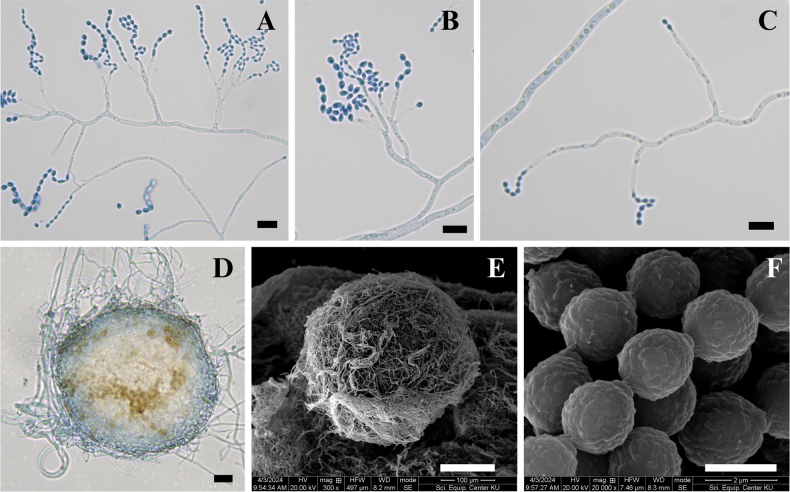
Morphological features of *Penicillium buranakarlianum* DMKU-RS5M3^T^. **A–C.** Conidia and conidiophores were rarely observed. Conidiophores are monoverticillate to diverticillate, hyaline, and smooth-walled. Phialides are ampulliform, borne singly or in groups of up to four per conidiophore, smooth-walled, and hyaline; **D, E.** Abundant sclerotia were produced; **F.** Under scanning electron microscopy, conidia appear subglobose to ovoid with delicate surface ornamentation consisting of very short ridges. Sexual morph was not observed. Scale bars: 15 µm (**A–D**); 10 µm (**E**); 2 µm (**F**).

On CYA at 25 °C after 7 days, colonies were slightly umbonate with undulate margins; mycelium yellowish white (4A2) at the centre and white (1A1) toward the margins, velvety in texture; sporulation very sparse, conidia *en masse* not determined; sclerotia absent; exudate absent; no soluble pigments produced; reverse pale yellow (4A3) (Fig. 8A). On MEA at 25 °C after 7 days, colonies were slightly umbonate with undulate margins; mycelium white (1A1), velvety in texture; sporulation very sparse, conidia *en masse* not determined; sclerotia abundant, white (1A1); exudate hyaline; no soluble pigments produced; reverse champagne (4A4) (Fig. 8B). On CY20S at 25 °C after 7 days, colonies were slightly raised with undulate margins; mycelium white (1A1), velvety in texture; sporulation very sparse, conidia *en masse* not determined; sclerotia and exudate absent; no soluble pigments produced; reverse pale yellow (4A3) (Fig. 8C). On CYAS at 25 °C after 7 days, colonies were slightly raised with filiform margins; mycelium white (1A1), velvety in texture; sporulation very sparse, conidia *en masse* not determined; sclerotia and exudate absent; no soluble pigments produced; reverse pale yellow (4A3) (Fig. 8D). On CZ at 25 °C after 7 days, colonies were umbonate with undulate margins; mycelium white (1A1), velvety in texture; sporulation very sparse, conidia *en masse* not determined; sclerotia and exudate absent; no soluble pigments produced; reverse yellowish white (4A2) (Fig. 8E). On OA at 25 °C after 7 days, colonies were plain with entire margins; mycelium white (1A1), granular in texture; sporulation absent to very sparse; sclerotia abundant, white (1A1); exudate absent; no soluble pigments produced; reverse white (1A1) (Fig. 8F). On CREA at 25 °C after 7 days, colonies showed poor growth; no acid production observed (Fig. 8G). On PDA at 25 °C after 7 days, colonies were slightly umbonate with undulate margins; mycelium white (1A1), granular in texture; sporulation very sparse, conidia *en masse* not determined; sclerotia abundant, white (1A1); exudate absent; no soluble pigments produced; reverse pale yellow (3A3) at the centre, white (1A1) at the margins (Fig. 8H). On CYA at 30 °C after 7 days, colonies were slightly umbonate with undulate margins; mycelium white (1A1), velvety in texture; sporulation very sparse, conidia *en masse* not determined; sclerotia and exudate absent; no soluble pigments produced; reverse pale yellow (4A3) (Fig. 8I). On CYA at 37 °C after 7 days, no growth were observed (Fig. 8J). Asexual spores were rarely observed, while abundant sclerotia were produced on PDA and MEA (Fig. 9D, E).

##### Micromorphology.

Conidia and Conidiophores were rarely produced (Fig. 9A–C). Abundant Sclerotia were produced (Fig. 9D, E). Conidiophores were monoverticillate to diverticillate, hyaline, smooth-walled, measuring 8.0–93.5 × 2.5–5.0 µm. Phialides ampulliform, borne singly or in groups of up to four per conidiophore, smooth-walled, hyaline, measuring 8.0–19.0 × 3.0–5.0 µm. Conidia were subglobose to ovoid, measuring 3.0–6.0 × 2.5–5.0 µm. Under scanning electron microscopy, conidia appear with delicate surface ornamentation consisting of very short ridges (Fig. 9F). Sexual morph was not observed.

##### Habitat and distribution.

Mangrove sediment; only known from Chanthaburi Province, Thailand.

##### Notes.

*Penicillium buranakarlianum* is classified in section Lanata-Divaricata, series *Janthinella*. Phylogenetic analyses resolved it in a clade with *P. ehrlichii* (Stolk and Scott 1967), *P. melanosporum*, *P. siccitolerans* (Rodriguez-Andrade et al. 2021) and *P. meloforme* (Udagawa and Horie 1973). However, sexual morphs (cleistothecia) are absent in *P. buranakarlianum* but present in *P. ehrlichii* and *P. meloforme*. Moreover *P. melanosporum* further differs by producing conidia that are olive-green to dark brown and enveloped by a dark brown sheath at maturity. Although both *P. buranakarlianum* and *P. siccitolerans* produce abundant sclerotia, the latter can grow at temperatures of up to 40 °C, whereas *P. buranakarlianum* is unable to grow at 37 °C.

### ﻿Antibacterial activity against pathogenic bacteria

**Figure 10.**
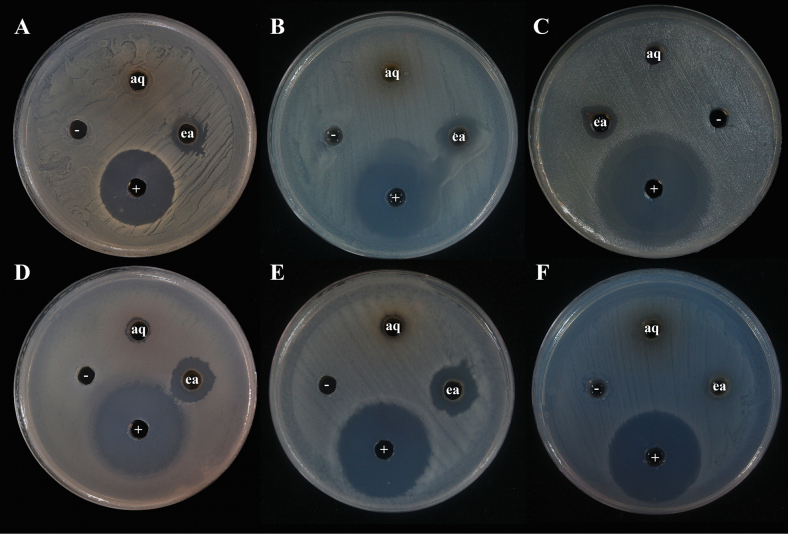
Antibacterial activity of *Penicillium chanthaburiense* DMKU-SS6M3^T^. **A.** Methicillin resistance *Staphylococcus aureus* (Clinical isolate); **B.***Staphylococcus aureus* ATCC 25923; **C.***Staphylococcus aureus* ATCC 29213; **D.***Bacillus subtilis* ATCC 6051; **E.***Bacillus cereus* ATCC 11778; **F.***Escherichia coli* ATCC 25922. “ea” indicates the crude extract from the ethyl acetate phase; “aq” indicates the crude extract from the aqueous phase; “+” denotes the positive control (streptomycin, 10 mg/mL); “−” denotes the negative control (20% DMSO).

The antibacterial activity of the *Penicillium* species is presented in Table 2. The ethyl acetate fraction of the crude extract from *P. chanthaburiense* DMKU-SS6M3^T^ exhibited inhibitory activity against Gram-positive bacteria, particularly *S. aureus*, *B. subtilis*, and *B. cereus* (Fig. 10). Notably, it also showed activity against MRSA. However, this extract showed no activity against any of the tested Gram-negative bacteria. Furthermore, the aqueous phase obtained after the ethyl acetate extraction of *P. chanthaburiense* DMKU-SS6M3^T^ did not exhibit any antibacterial activity (Fig. 10). In addition, the ethyl acetate and aqueous fractions from *P. danzhouense* DMKU-SS7M2 and *P. buranakarlianum* DMKU-RS5M3^T^ showed no antibacterial activity against any of the tested bacterial strains.

**Table 2.** Antibacterial activity of ethyl acetate extract from three *Penicillium* species isolated from mangrove sediment in Thailand.

Bacterial strains	Size of inhibition zone (mm.)		
	*P. chanthaburiense* DMKU-SS6M3^T^	*P. danzhouense* DMKU-SS7M2	*P. buranakarlianum* DMKU-RS5M3^T^
Methicillin resistance *Staphylococcus aureus* (Clinical isolate)	**12.79 ± 0.57**	0.00 ± 0.00	0.00 ± 0.00
*Staphylococcus aureus* ATCC 25923	**14.70 ± 0.33**	0.00 ± 0.00	0.00 ± 0.00
*Staphylococcus aureus* ATCC 29213	**12.50 ± 0.19**	0.00 ± 0.00	0.00 ± 0.00
*Bacillus subtilis* ATCC 6051	**16.50 ± 0.30**	0.00 ± 0.00	0.00 ± 0.00
*Bacillus subtilis* 7988 (Clinical isolate)	**14.42 ± 0.56**	0.00 ± 0.00	0.00 ± 0.00
*Bacillus cereus* ATCC 11778	**16.12 ± 0.07**	0.00 ± 0.00	0.00 ± 0.00
*Escherichia coli* ATCC 25922	0.00 ± 0.00	0.00 ± 0.00	0.00 ± 0.00
*Escherichia coli* O157:H7 (Clinical isolate)	0.00 ± 0.00	0.00 ± 0.00	0.00 ± 0.00
*Pseudomonas aeruginosa* ATCC 27853	0.00 ± 0.00	0.00 ± 0.00	0.00 ± 0.00
*Klebsiella pneumoniae* ATCC 70063	0.00 ± 0.00	0.00 ± 0.00	0.00 ± 0.00
*Shigella enterotidis* (Clinical isolate)	0.00 ± 0.00	0.00 ± 0.00	0.00 ± 0.00
*Salmonella enterica* serotype Typhi (Clinical isolate)	0.00 ± 0.00	0.00 ± 0.00	0.00 ± 0.00
*Vibrio cholerae* (Clinical isolate)	0.00 ± 0.00	0.00 ± 0.00	0.00 ± 0.00

## ﻿Discussion

The genus *Penicillium* is one of the most morphologically diverse and ecologically widespread fungal genera, encompassing taxa that thrive across a variety of terrestrial and aquatic ecosystems (Houbraken et al. 2020). Mangrove ecosystems, characterised by high salinity, tidal fluctuation, and organic-rich substrates, have emerged as promising but underexplored habitats for discovering novel fungal diversity (Norphanphoun et al. 2019; Apurillo et al. 2025,). This study introduces two new species of *Penicillium* (*P. chanthaburiense*, and *P. buranakarlianum*) and first record *P. danzhouense* recovered from mangrove sediment samples collected at the King Rama IX International Mangrove Botanical Garden in eastern Thailand.

These findings underscore the high and yet underestimated diversity of *Penicillium* species inhabiting mangrove ecosystems. Previous studies have suggested that saline, organic-rich environments such as mangroves may serve as specialised niches driving fungal diversification (Jones et al. 2011; Lee et al. 2019). The consistent recovery of two new taxa from multiple sampling sites, particularly sample sites 6 and 7, further suggests that these species are not atypical or sporadic occurrences but may play active ecological roles in the microbial community in mangrove sediment.

Previously, only two species in section Exilicaulis (*P. laeve* and *P. terrae*) had been reported from Thailand, both isolated from forest soil (Ando et al. 1998; Thitla et al. 2025). The identification of *P. chanthaburiense* from mangrove sediment not only expands the known habitat range of this section but also represents the third species of *Exilicaulis* recorded in the country. Similarly, the description of *P. buranakarlianum* in section Lanata-Divaricata adds a third species to this section in Thailand, following *P. singorense* from house dust (Visagie et al. 2014) and *P. chaingmaiense* from a forest dump site (Thitla et al. 2025).

In conclusion, the description of *P. chanthaburiense* and *P. buranakarlianum* adds to the growing inventory of mangrove-associated fungi and highlights the ecological breadth of *Penicillium* in saline habitats. Further exploration of these ecosystems will undoubtedly continue to yield novel fungal lineages and refine our understanding of the evolution and taxonomy of *Penicillium*.

Among these three *Penicillium* species, only *P. chanthaburiense* DMKU-SS6M^T^ showed notable antibacterial activity, specifically against several Gram-positive bacteria, including *S. aureus*, *B. subtilis* and *B. cereus*, when extracted with ethyl acetate. In contrast, aqueous extracts of *P. chanthaburiense* and all extracts of *P. danzhouense* and *P. buranakarlianum* showed no activity. These findings highlight not only the strain-specific nature of antibacterial activity but also the importance of the extraction method. Ethyl acetate is a semi-polar organic solvent capable of extracting a wide range of non-polar to moderately polar secondary metabolites, including many bioactive compounds such as polyketides and alkaloids. In contrast, aqueous extractions are more likely to capture polar compounds, which may lack antibacterial properties or may not effectively penetrate bacterial membranes. The observed selectivity toward Gram-positive bacteria further supports this, as their cell walls consist of a thick peptidoglycan layer without an outer membrane, making them more accessible to many hydrophobic or amphipathic compounds. On the other hand, Gram-negative bacteria possess an outer membrane rich in lipopolysaccharides that can restrict the entry of such compounds, potentially explaining the lack of activity. Collectively, these results underscore the potential of *P. chanthaburiense* as a promising source of bioactive metabolites, particularly against Gram-positive pathogens, and emphasize the critical role of the extraction strategy in bioactivity-guided screening.

The ability of *P. chanthaburiense* to inhibit MRSA is of particular interest given the global concern over antimicrobial resistance. Mangrove-derived fungi have been increasingly recognised as a valuable source of novel bioactive compounds due to their adaptation to stressful, nutrient-variable environments (Xu et al. 2015). The findings in this study suggest that *P. chanthaburiense* may contribute to this potential and underscore the utility of exploring underexplored habitats such as mangrove sediments in the search for new antimicrobial agents.

Future studies should aim to isolate and chemically characterise the specific secondary metabolites responsible for the observed antibacterial activity. Identifying and elucidating the structure of these compounds could provide valuable information for the development of new antibiotics, particularly against resistant bacterial strains. The discovery of bioactivity in *P. chanthaburiense* thus opens an exciting avenue for future research at the interface of fungal taxonomy, natural product chemistry, and drug discovery.

## Supplementary Material

XML Treatment for Penicillium chanthaburiense

XML Treatment for Penicillium danzhouense

XML Treatment for Penicillium buranakarlianum

## ﻿Additional information

### Use of AI

No use of AI was reported.

### Author ORCIDs

Wanchat Sirisarn https://orcid.org/0000-0002-9290-0238

Jui-Yu Chou https://orcid.org/0000-0002-4770-3702

Piyangkun Lueangjaroenkit https://orcid.org/0000-0002-1362-2971

### Data availability

All of the data that support the findings of this study are available in the main text or Supplementary Information.

## Supplementary materials

Supplementary material 1 List of *Penicillium* species used in this study Data type docx https://binary.pensoft.net/file/1498077This dataset is made available under the Open Database License (http://opendatacommons.org/licenses/odbl/1.0/). The Open Database License (ODbL) is a license agreement intended to allow users to freely share, modify, and use this Dataset while maintaining this same freedom for others, provided that the original source and author(s) are credited.Vassana Supapongsri, Ananya Sahathippayakul, Wanchat Sirisarn, Mohit Chand, Jui-Yu Chou, Piyangkun Lueangjaroenkit

Supplementary material 2 Single-gene phylogenies Data type docx Explanation note Single gene phylogenetic tree of Penicillium in section Lanata-Divaricata. https://binary.pensoft.net/file/1498078This dataset is made available under the Open Database License (http://opendatacommons.org/licenses/odbl/1.0/). The Open Database License (ODbL) is a license agreement intended to allow users to freely share, modify, and use this Dataset while maintaining this same freedom for others, provided that the original source and author(s) are credited.Vassana Supapongsri, Ananya Sahathippayakul, Wanchat Sirisarn, Mohit Chand, Jui-Yu Chou, Piyangkun Lueangjaroenkit
