# Supplementary material for: ﻿Two new species of Penicillium (Eurotiales, Aspergillaceae) and the first record of P. danzhouense from mangrove sediment in Thailand, with notes on antibacterial activity
Source: MycoKeys. 2025 Dec 22;126:213–38. doi: 10.3897/mycokeys.126.172211 (PMC12750103; doi:10.3897/mycokeys.126.172211)
Supplement: Supplementary material 1 — List of Penicillium species used in this study [file mycokeys-126-213-s001.docx]

**Table S1.** List of *Penicillium* species used in this study

| **Section** | **Series** | **Species** | **Strains** | **Substrate/Host** | **Locality** | **GenBank Accessions** | | | | |
| --- | --- | --- | --- | --- | --- | --- | --- | --- | --- | --- |
|  |  |  |  |  |  | ***ITS*** | ***BenA*** | ***CaM*** | ***RPB2*** |  |
| *Exilicaulis* | *Lapidosa* | *P. velutinum* | CBS  250.32 = NRRL 2069 | Sputum from man | The  Netherlands | AF033448 | JX141170 | MT478037 | KP064682 |  |
| *Exilicaulis* | *Lapidosa* | *P. maclennaniae* | CBS  198.81 = DAR 35238 | Rhizoplane of *Gahnia*  *radula* | Australia | KC411689 | KJ834468 | KP016791 | KP064648 |  |
| *Exilicaulis* | *Lapidosa* | *P. hemitrachum* | CBS 139134 = CV 2845 | Air | South Africa | FJ231003 | JX141048 | JX157526 | KP064642 |  |
| *Exilicaulis* | *Lapidosa* | *P. smithii* | CBS 276.83 | Secale cereale | Spain | KC411723 | KJ834492 | KP016806 | JN406589 |  |
| *Exilicaulis* | *Lapidosa* | *P. xanthomelinii* | CBS  139163 = CV 1677 | Soil | South Africa | JX140921 | JX141120 | JX141120 | KP064683 |  |
| *Exilicaulis* | *Lapidosa* | *P. aotearoae* | CBS  140999 = KAS 3088 | Clay | New Zealand | KT887874 | KT887835 | KT887796 | MN969174 |  |
| *Exilicaulis* | *Lapidosa* | *P. melinii* | CBS 218.30 = ATCC 10469 | Forest soil | USA | AF033449 | KJ834471 | KP016792 | JN406613 |  |
| *Exilicaulis* | *Lapidosa* | *P. diabolicalicense* | CBS 140967 = KAS 1726 | Beneath moss and  *Nothofagus* | New Zealand | KT887840 | KT887801 | KT887762 | MN969175 |  |
| *Exilicaulis* | *Lapidosa* | *P. atrosanguineum* | CBS 380.75 | Seeds of *Triticum* | Czech | JN617706 | KJ834435 | KP016771 | JN406557 |  |
| *Exilicaulis* | *Lapidosa* | *P. raciborskii* | CBS 224.28 = NRRL 2150 | Soil under conifer | Poland | AF033447 | JX141069 | KP016800 | JN406607 |  |
| *Exilicaulis* | *Lapidosa* | *P. burgense* | CBS 325.89 | Soil | Spain | KC411736 | KJ834437 | KP016772 | JN406572 |  |
| *Exilicaulis* | *Lapidosa* | *P. terrenum* | CBS 313.67 | Soil | South Africa | AM992111 | KJ834496 | KP016808 | JN406577 |  |
| *Exilicaulis* | *Lapidosa* | *P.lapidosum* | CBS 343.48 | Canned blueberries | USA | MN431392 | KJ834465 | FJ530984 | JN121500 |  |
| *Exilicaulis* | *Lapidosa* | *P. namyslowskii* | CBS 353.48 = NRRL 1070 | Soil under *Pinus* | Poland | AF033463 | JX141067 | KP016795 | JF417430 |  |
| *Exilicaulis* | *Corylophila* | *P. momoii* | CBS  139157 = CV1015 | Mite from *Protea repens* | South Africa | JX140895 | JX141073 | JX157479 | KP064673 |  |
| *Exilicaulis* | *Corylophila* | *P.consobrinum* | CBS  139144 = CV547 | Soil | South Africa | JX140888 | JX141135 | JX157453 | KP064619 |  |
| *Exilicaulis* | *Corylophila* | *P. rubefaciens* | CBS 145.83 | Sandy soil under *Pinus* sp. | Spain | KC411677 | KJ834487 | KP016804 | JN406627 |  |
| *Exilicaulis* | *Corylophila* | *P. subturcoseum* | CBS 139132 = CV 2835 | Soil | South Africa | FJ231006 | JX141161 | JX157532 | KP064674 |  |
| *Exilicaulis* | *Corylophila* | *P. repensicola* | CBS  139160 = CV1495 | Bract from *Protea repens* | South Africa | JX140893 | JX141150 | JX157490 | KP064660 |  |
| *Exilicaulis* | *Corylophila* | *P. pagulum* | CBS 139166 = CV 2224 | Bract from *Protea repens* | South Africa | JX140898 | JX141070 | JX157519 | KP064655 |  |
| *Exilicaulis* | *Corylophila* | *P. cravenianum* | CBS  139138 = CV 92 | Soil | South Africa | JX140900 | JX141076 | JX157418 | KP064636 |  |
| *Exilicaulis* | *Corylophila* | *P. corylophilum* | CBS  312.48 = NRRL  802 | Unknown | Unknown | AF033450 | JX141042 | KP016780 | KP016780 |  |
| *Exilicaulis* | *Corylophila* | *P. atrolazulinum* | CBS  139136 = CV 55 | Air | South Africa | JX140913 | JX141077 | JX157416 | KP064575 |  |
| *Exilicaulis* | *Corylophila* | *P. fagi* | CBS  689.77 = CCMF-696 = IJFM 3049 = IMI 253806 | Fallen leaf (*Fagus sylvatica*) on andosol soi | Spain | AF481124 | KJ834449 | KP016784 | JN406540 |  |
| *Exilicaulis* | *Restricta* | *P. arabicum* | CBS 414.69 | Soil | Syria | KC411758 | KP016750 | KP016770 | KP064574 |  |
| *Exilicaulis* | *Restricta* | *P. katangense* | CBS 247.67 = NRRL 5182 | Soil | Congo | AF033458 | KP016757 | KP016788 | KP064646 |  |
| *Exilicaulis* | *Restricta* | *P. cinereoatrum* | CBS 222.66 | Forest soil | Ukraine | KC411700 | KJ834442 | KP125335 | JN406608 |  |
| *Exilicaulis* | *Restricta* | *P. heteromorphum* | CBS 226.89 | Soil | China | KC411702 | KJ834455 | KP016786 | JN406605 |  |
| *Exilicaulis* | *Restricta* | *P. kurssanovii* | CBS 625.67 = NRRL 3381 | Maize-eld soi | Ukraine | EF422849 | KP016758 | KP016789 | KP064647 |  |
| *Exilicaulis* | *Restricta* | *P. chalabudae* | CBS 219.66 | Soil | Ukraine | KP016811 | KP016748 | KP016767 | KP064572 |  |
| *Exilicaulis* | *Restricta* | *P. restrictum* | CBS  367.48 = NRRL 1748 | Soil | Honduras | AF033457 | KJ834486 | KP016803 | JN121506 |  |
| *Exilicaulis* | *Restricta* | *P. meridianum* | CBS  314.67 = NRRL 5814 | Garden soil | USA | AF033451 | KJ834472 | KP016794 | JN406576 |  |
| *Exilicaulis* | *Restricta* | *P. philippinense* | CBS 623.72 | Soil from pine forest | Philippines | KC411770 | KJ834482 | KP016799 | JN406543 |  |
| *Exilicaulis* | *Alutacea* | *P. alutaceum* | CBS 317.67 = ATCC 18542 | Soil | South Africa | AF033454 | KJ834430 | KP016768 | JN121489 |  |
| *Exilicaulis* | *Alutacea* | *P. decumbens* | CBS 230.81 | Unknown | USA | AY157490 | KJ834446 | KP016782 | JN406601 |  |
| *Exilicaulis* | *Citreonigra* | *P. cinerascens* | NRRL 748 | Unknown | Unknown | AF033455 | JX141041 | JX157405 | MN969112 |  |
| *Exilicaulis* | *Citreonigra* | *P. fundyense* | KAS 2174 | Bay of Fundy | Canada | KT887853 | KT887814 | KT887775 | MN969176 |  |
| *Exilicaulis* | *Citreonigra* | *P. citreonigrum* | CBS  258.29 = NRRL 761 | Rotting stem | Belgium | AF033456 | EF198621 | EF198628 | JN121474 |  |
| *Exilicaulis* | *Citreonigra* | *P. citreosulfuratum* | MI 92228 = DTO 290-I4 | Unknown | Unknown | KP016814 | KP016753 | KP064615 | KP016777 |  |
| *Exilicaulis* | *Erubescentia* | *P. guttulosum* | CBS 141171 = NRRL 907 | Soil | USA | HQ646592 | HQ646576 | HQ646587 | MG386247 |  |
| *Exilicaulis* | *Erubescentia* | *P. rubidurum* | CBS 609.73 = NRRL 6033 | Soil | Papua New Guinea | AF033462 | HQ646574 | HQ646585 | JN406545 |  |
| *Exilicaulis* | *Erubescentia* | *P. pimiteouiense* | CBS 102479 = NRRL  25542 | Kidney epithelial cell culture flask | USA | AF037431 | HQ646569 | HQ646580 | JN406650 |  |
| *Exilicaulis* | *Erubescentia* | *P. menonorum* | NRRL 50410 | Garden soil | USA | HQ646591 | HQ646573 | HQ646584 | KF900194 |  |
| *Exilicaulis* | *Erubescentia* | *P. parvum* | CBS 359.48 = NRRL 2095 | Soil | Nicaragua | AF033460 | HQ646568 | KF900173 | JN406559 |  |
| *Exilicaulis* | *Erubescentia* | *P. vinaceum* | CBS  389.48 = NRRL 739 | Soil | USA | AF033461 | HQ646575 | HQ646586 | JN406555 |  |
| *Exilicaulis* | *Erubescentia* | *P. erubescens* | CBS 318.67 = NRRL 6223 | Nursery soil | South Africa | AF033464 | HQ646566 | EU427281 | JN121490 |  |
| *Exilicaulis* | *Erubescentia* | *P. nepalense* | CBS  203.84 | Rice soil | Nepal | KC411692 | KJ834474 | KP016796 | JN121453 |  |
| *Exilicaulis* | *Erubescentia* | *P. catenatum* | CBS 352.67 | Desert soil | South Africa | KC411754 | KJ834438 | KP016774 | JN121504 |  |
| *Exilicaulis* | *Erubescentia* | *P. canis* | NRRL 62798 | Ilial bone lesion in Rhodesian ridgeback dog | USA | KJ511291 | KF900167 | KF900177 | KF900196 |  |
| *Exilicaulis* | *Erubescentia* | *P. striatisporum* | CBS 705.68 = NRRL 26877 | Leaf litter of *Acacia karroo* | South Africa | AF038938 | MN969401 | KP016807 | JN406538 |  |
| *Exilicaulis* | *Erubescentia* | *P. hermansii* | DTO 079-D5 = CBS 124296 | Mushroom compost with smoky mould | The Netherlands | MG333472 | MG386214 | MG386229 | MG386242 |  |
| *Exilicaulis* | *Erubescentia* | *P. dimorphosporum* | CBS 456.70 = NRRL 5207 | Mangrove swamp soil | Australia | AF081804 | KJ834448 | KP016783 | JN121517 |  |
| *Exilicaulis* | *Erubescentia* | *P. parvofructum* | FMR  15047 = CBS 141690 | Soil | Spain | LT559091 | LT627645 | LT627646 | MN969197) |  |
| *Exilicaulis* | *Erubescentia* | *P. laeve* | CBS 136665 = DTO 270-G8 | Forest soil | Thailand | KF667369 | KF667365 | KF667367 | KF667371 |  |
| *Exilicaulis* | *Erubescentia* | *P. ovatum* | CBS 136664 = DTO 270-G7 | Forest soil under litter of *Pinus caribaea* | Malayasia | KF667370 | KF667366 | KF667368 | KF667372 |  |
| *Exilicaulis* | *Erubescentia* | *P.danzhouense* | CGMCC 3.25204 | Sediment | China | ON563150 | ON231295 | ON470838 | n.a. |  |
| *Exilicaulis* | *Erubescentia* | *P. amapaense* | *INPA-AP07* | Sediments from the Amazon River | Brazil | OL764382 | OL782590 | OL782584 | ON854925 |  |
| *Exilicaulis* | *Erubescentia* | *P. labradorum* | DI19-20 | Labrador retriever with disseminated fungal disease | USA | MK881918 | MK887898 | MK887899 | *MK887900* |  |
| *Exilicaulis* | *Erubescentia* | *P. tenue* | CGMCC 3.25205 | Tidal flat sediments | China | ON563151 | ON231296 | ON470839 | ON470842 |  |
| *Exilicaulis* | *Erubescentia* | *P. terrae* | MBSZU 24-008 | Soil in the forest dump-sites | Thailand | PV036869 | PV054939 | PV054943 | PV036876 |  |
| *Exilicaulis* | *Erubescentia* | *P. vallebormidaense* | DTO 402-H5 | Compost | Italy | MT316359 | MW115862 | MW115863 | MW115864 |  |
| ***Exilicaulis*** | ***Erubescentia*** | ***P. chanthaburiense* ^T^** | **SS6P3** | **Mangrove soil sediment** | **Thailand** | **LC780059** | **LC796777** | **LC796778** | **LC871398** |  |
| ***Exilicaulis*** | ***Erubescentia*** | ***P. chanthaburiense*** | **SS11P5** | **Mangrove soil sediment** | **Thailand** | **LC796788** | **LC871399** | **LC796790** | **LC871400** |  |
| ***Exilicaulis*** | ***Erubescentia*** | ***P. chanthaburiense*** | **SS9P12** | **Mangrove soil sediment** | **Thailand** | **LC796785** | **LC796786** | **LC796787** | **LC871397** |  |
| ***Exilicaulis*** | ***Erubescentia*** | ***P. chanthaburiense*** | **SS7M1** | **Mangrove soil sediment** | **Thailand** | **LC796782** | **LC796783** | **LC796784** | **LC871401** |  |
| ***Exilicaulis*** | ***Erubescentia*** | ***P. chanthaburiense*** | **RS6P10** | **Mangrove soil sediment** | **Thailand** | **LC780057** | **LC796773** | **LC796774** | **LC871403** |  |
| ***Exilicaulis*** | ***Erubescentia*** | ***P. chanthaburiense*** | **RS7P10** | **Mangrove soil sediment** | **Thailand** | **LC796779** | **LC796780** | **LC796781** | **LC871404** |  |
| ***Exilicaulis*** | ***Erubescentia*** | ***P. danzhouense*** | **SS7M2** | **Mangrove soil sediment** | **Thailand** | **LC796798** | **LC871410** | **LC796800** | **LC871411** |  |
| ***Exilicaulis*** | ***Erubescentia*** | ***P. danzhouense*** | **SS11P3** | **Mangrove soil sediment** | **Thailand** | **LC796812** | **LC796813** | **LC796814** | **LC871409** |  |
| ***Exilicaulis*** | ***Erubescentia*** | ***P. danzhouense*** | **SS7P3** | **Mangrove soil sediment** | **Thailand** | **LC796801** | **LC796802** | **LC796803** | **LC871407** |  |
| ***Exilicaulis*** | ***Erubescentia*** | ***P. danzhouense*** | **SS6M3** | **Mangrove soil sediment** | **Thailand** | **LC796791** | **LC796792** | **LC796793** | **LC871408** |  |
| ***Exilicaulis*** | ***Erubescentia*** | ***P. danzhouense*** | **SS9M4** | **Mangrove soil sediment** | **Thailand** | **LC796804** | **LC796805** | **LC796806** | **LC871412** |  |
| ***Exilicaulis*** | ***Erubescentia*** | ***P. danzhouense*** | **SS12P2** | **Mangrove soil sediment** | **Thailand** | **LC871405** | **LC796816** | **LC796817** | **LC871406** |  |
| **Citrina* | *Euglauca* | *P. anatolicum (outgroup)* | CBS 479.66 | Unknown | Unknown | AF033425 | JN606849 | JN606571 | JN606593 |  |
| *Lanata-Divaricata* | *Dalearum* | *P. abidjanum* | CBS 246.67 | Savannah soil | Ivory Coast | GU981582 | GU981650 | MN969234 | JN121469 |  |
| *Lanata-Divaricata* | *Dalearum* | *P. albineum* | URM 8918 | Cave sediment | Brazil | PP505396 | PP806627 | PP806632 | PP942619 |  |
| *Lanata-Divaricata* | *Dalearum* | *P. amphipolaria* | CBS 140997  = KAS 2555 | Soil | Antarctica | KT887872 | KT887833 | KT887794 | MN969177 |  |
| *Lanata-Divaricata* | *Dalearum* | *P. aureosclerotiorum* | URM 8919 | Cave sediment | Brazil | PP505393 | PP806628 | PP806633 | PP942620 |  |
| *Lanata-Divaricata* | *Dalearum* | *P. ausonanum* | FMR 16948 | Fluvial sediment | Spain | LR655808 | LR655809 | LR655810 | LR655811 |  |
| *Lanata-Divaricata* | *Dalearum* | *P. austrosinense* | NN072318 | Acidic soil | China | KY495007 | KY495116 | MN969328 | KY495061 |  |
| *Lanata-Divaricata* | *Dalearum* | *P. carajasense* | URM 8917 | Cave sediment | Cave sediment | PP505390 | PP806626 | PP806631 | PP942618 |  |
| *Lanata-Divaricata* | *Dalearum* | *P. daleae* | CBS 211.28 | Soil under conifer | Poland | GU981583 | GU981649 | MN969251 | KF296427 |  |
| *Lanata-Divaricata* | *Dalearum* | *P. griseopurpureum* | CBS 406.65 | Soil under *Pinus* | UK | KF296408 | KF296467 | MN969261 | KF296431 |  |
| *Lanata-Divaricata* | *Dalearum* | *P. jianfenglingense* | NN072384 | Acidic soil | China | KY495016 | KY495125 | MN969334 | KY495069 |  |
| *Lanata-Divaricata* | *Dalearum* | *P. limpopoense* | CMW 59580  = CN 154A5 | Litchi | South Africa | MK450722 | MK451261 | MK451651 | MK450854 |  |
| *Lanata-Divaricata* | *Dalearum* | *P. parauapebasense* | URM 8916 | Cave sediment | Brazil | PP814733 | PP806625 | PP389046 | PP94261 |  |
| *Lanata-Divaricata* | *Dalearum* | *P. pauciramulum* | CGMCC 3.25164  = CS04-09 | Ant hole soil | China | OQ870726 | OR051111 | OR051288 | OR051457 |  |
| *Lanata-Divaricata* | *Dalearum* | *P. penarojense* | CBS 113178 | Forest leaf litter | Colombia | GU981570 | GU981646 | MN969287 | KF296450 |  |
| *Lanata-Divaricata* | *Dalearum* | *P. rubriannulatum* | NN072456  = CBS 144641 | Acidic soil | China | KY495029 | KY495138 | MN969336 | KY49508 |  |
| *Lanata-Divaricata* | *Dalearum* | *P. singorense* | CBS 138214 | House dust | Thailand | KJ775674 | KJ775167 | KJ775403 | MN969138 |  |
| *Lanata-Divaricata* | *Dalearum* | *P. stangiae* | URM 8347 | Forest soil | Brazil | MW648590 | MW646388 | MW646390 | MW646392 |  |
| *Lanata-Divaricata* | *Dalearum* | *P. vanderhammenii* | CBS 126216 | Forest leaf litter | Colombia | GU981574 | GU981647 | MN969308 | KF296458 |  |
| *Lanata-Divaricata* | *Dalearum* | *P. viridissimum* | NN072081  = CBS 144484 | Acidic soil | China | KY495004 | KY495113 | MN969339 | KY495059 |  |
| *Lanata-Divaricata* | *Dalearum* | *P. zonatum* | CBS992.72 | Coastal marsh soil | USA | GU981581 | GU981651 | MN969315 | KF296461 |  |
| *Lanata-Divaricata* | *Janthinella* | *P. ashbyae* | MST FP22310 | Soil | Australia | OR731310 | OR737776 | OR737766 | OR737771 |  |
| *Lanata-Divaricata* | *Janthinella* | *P. brefeldianum* | CBS 235.81  = NRRL 710 | Human alimentary tract | Unknown | AF033435 | GU981623 | EU021683 | KF296421 |  |
| *Lanata-Divaricata* | *Janthinella* | *P. caperatum* | CBS 443.75 | Soil | Australia | KC411761 | GU981660 | MN969242 | KF296422 |  |
| *Lanata-Divaricata* | *Janthinella* | *P. coeruleum* | CBS 141.45 | Unknown | Unknown | GU981606 | GU981655 | MN969247 | KF296425 |  |
| *Lanata-Divaricata* | *Janthinella* | *P. cremeogriseum* | CBS 223.66 | Forest soil | Ukraine | GU981586 | GU981624 | MN969250 | KF296426 |  |
| *Lanata-Divaricata* | *Janthinella* | *P. curticaule* | CBS 135127  = CV 2842 = CV 188 | Soil | South  Africa | FJ231021 | JX091526 | JX141536 | KF296417 |  |
| *Lanata-Divaricata* | *Janthinella* | *P. donggangicum* | AS3.15900 | Soil of tidal flats | China | MW946996 | MZ004914 | MZ004918 | MW979253 |  |
| *Lanata-Divaricata* | *Janthinella* | *P. ehrlichii* | CBS 324.48 | Unknown | Poland | GU981578 | GU981652 | MN969253 | KF296428 |  |
| *Lanata-Divaricata* | *Janthinella* | *P. elleniae* | CBS 118135 | Forest leaf litter | Colombia | GU981612 | GU981663 | MN969254 | KF296429 |  |
| *Lanata-Divaricata* | *Janthinella* | *P. glaucoroseum* | DTO 225-E8 = CBS 138908 | Soil | USA | MN431390 | MN969383 | MN969257 | MN969119 |  |
| *Lanata-Divaricata* | *Janthinella* | *P. janthinellum* | CBS 340.48 | Soil | Nicaragua | GU981585 | GU981625 | MN969268 | JN121497 |  |
| *Lanata-Divaricata* | *Janthinella* | *P. javanicum* | CBS 341.48 | Root of *Camellia sinensis* | Indonesia | GU981613 | GU981657 | MN969269 | JN121498 |  |
| *Lanata-Divaricata* | *Janthinella* | *P. koreense* | CBS 141338T  = KACC  47721 = DTO 347-  C1 | Soil | South Korea | KJ801939 | KM000846 | MN969317 | MN969159 |  |
| *Lanata-Divaricata* | *Janthinella* | *P. levitum* | CBS 345.48 | Modelling clay | USA | GU981607 | GU981654 | MN969270 | KF296432 |  |
| *Lanata-Divaricata* | *Janthinella* | *P. limosum* | CBS 339.97 | Marine sediment | Japan | GU981568 | GU981621 | MN969271 | KF296433 |  |
| *Lanata-Divaricata* | *Janthinella* | *P. lineolatum* | CBS 188.77 | Soil from copse | Japan | GU981579 | GU981620 | MN969272 | KF296434 |  |
| *Lanata-Divaricata* | *Janthinella* | *P. ludwigii* | CBS 417.68 | Polished seed of *Oryza sativa* | Japan | KF296409 | KF296468 | MN969273 | KF296435 |  |
| *Lanata-Divaricata* | *Janthinella* | *P. malacosphaerulum* | CBS 135120  = CV  2855 | Soil | South  Africa | FJ231026 | JX091524 | JX141542 | KF296438 |  |
| *Lanata-Divaricata* | *Janthinella* | *P. melanosporum* | FMR 17424 | Soil | Spain | LR655192 | LR655196 | LR655200 | LR655204 |  |
| *Lanata-Divaricata* | *Janthinella* | *P. meloforme* | CBS 445.74 | Soil | Papua New Guinea | KC411762 | GU981656 | MN969276 | KF296440 |  |
| *Lanata-Divaricata* | *Janthinella* | *P. michoacanense* | FMR 17612 | Soil | Mexico | LR655194 | LR655198 | LR655202 | LR655206 |  |
| *Lanata-Divaricata* | *Janthinella* | *P. nordestinense* | URM 8423 | Pollen samples inside nests of  *Melipona scutellaris* | Brazil | OV265270 | OV265324 | OV265272 | OM927721 |  |
| *Lanata-Divaricata* | *Janthinella* | *P. ortum* | CBS 135669  = CV  102 | Soil | South  Africa | JX091427 | JX091520 | JX141551 | KF296443 |  |
| *Lanata-Divaricata* | *Janthinella* | *P. potchefstroomense* | CMW 59591 | Pear | South Africa | MK450727 | MK451229 | MK451662 | MK450865 |  |
| *Lanata-Divaricata* | *Janthinella* | *P. raperi* | CBS 281.58 = NRRL 2674 | Soil | UK | AF033433 | GU981622 | MN969291 | KF296453 |  |
| *Lanata-Divaricata* | *Janthinella* | *P. reticulisporum* | CBS 122.68  = NRRL 3447 | Soil | Japan | AF033437 | MN969394 | MN969293 | KF296454 |  |
| *Lanata-Divaricata* | *Janthinella* | *P. reverso-vinaceum* | URM 8967 | Cave sediment | Brazil | PP505387 | PP806630 | PP806635 | PP942622 |  |
| *Lanata-Divaricata* | *Janthinella* | *P. siccitolerans* | FMR 17381 | Soil | Spain | LR655193 | LR655197 | LR655201 | LR655205 |  |
| *Lanata-Divaricata* | *Janthinella* | *P. silvertonense* | CMW 59585 = CN153I2 | Onion | South Africa | MK450731 | MK451122 | MK451666 | MK450869 |  |
| *Lanata-Divaricata* | *Janthinella* | *P. soli* | KUMCC 18-0202 | Rhizosphere soil of *Quercus rubra* | China | MT152337 | MT161681 | MT178249 | MT384372 |  |
| *Lanata-Divaricata* | *Janthinella* | *P. tengii* | CGMCC 3.25179  = CS27-03 | Soil | China | OQ870735 | OR051120 | OR051297 | OR051465 |  |
| *Lanata-Divaricata* | *Janthinella* | *P. uruguayense* | FMR 14490 = CBS 143247 | Soil | Uruguay | LT904729 | LT904699 | LT904698 | MN969200 |  |
| *Lanata-Divaricata* | *Janthinella* | *P. yunnanense* | CBS 144485  = NN051336 | Acidic soil | China | KY494990 | KY495099 | MN969340 | KY495048 |  |
| *Lanata-Divaricata* | *Janthinella* | *P. chiangmaiense* | MBSZU 24-009 | Soil in the forest dump-sites | Thailand | PV036870 | PV054940 | PV054944 | PV036877 |  |
| ***Lanata-Divaricata*** | *Janthinella* | ***P. buranakarlianum*** | **RS12P5** | **Mangrove soil sediment** | **Thailand** | **LC796822** | **LC871420** | **LC871421** | **LC871422** |  |
| ***Lanata-Divaricata*** | *Janthinella* | ***P. buranakarlianum* ^T^** | **RS5M3** | **Mangrove soil sediment** | **Thailand** | **LC796823** | **LC871416** | **LC871417** | **LC871418** |  |
| ***Lanata-Divaricata*** | *Janthinella* | ***P. buranakarlianum*** | **RS6P1** | **Mangrove soil sediment** | **Thailand** | **LC796824** | **LC871413** | **LC871414** | **LC871415** |  |
| ***Lanata-Divaricata*** | *Janthinella* | ***P. buranakarlianum*** | **SS7P5** | **Mangrove soil sediment** | **Thailand** | **LC796818** | **LC796819** | **LC796820** | **LC871419** |  |
| *Lanata-Divaricata* | *Rolfsiorum* | *P. annulatum* | CBS 135126  = CV  0037 | Air | South  Africa | JX091426 | JX091514 | JX141545 | KF296410 |  |
| *Lanata-Divaricata* | *Rolfsiorum* | *P. bissetti* | KAS 1951  = CBS 140972 | Soil from Spruce  fores | Canada | JX091426 | JX091514 | JX141545 | KF296410 |  |
| *Lanata-Divaricata* | *Rolfsiorum* | *P. camponotum* | KAS 2177  = CBS 140982 | Carpenter ants (*Camponotus*  *pennsylvanicus*) | Canada | KT887855 | KT887816 | KT887777 | MN969179 |  |
| *Lanata-Divaricata* | *Rolfsiorum* | *P. coffeatum* | CGMCC 3.25152  = CS10-15 | Soil | China | OQ870815 | OR051121 | OR051298 | OR051466 |  |
| *Lanata-Divaricata* | *Rolfsiorum* | *Pe. excelsum* | DTO 357-D7 | Nut shell of *Bertholletia excelsa* | Brazil | (KR815341 | KP691061 | KR815342 | MN969166 |  |
| *Lanata-Divaricata* | *Rolfsiorum* | *P. flaviroseum* | NN072483  = CBS 144479 | Acidic soil | China | KY495032 | KY495141 | /MN969329 | KY495083 |  |
| *Lanata-Divaricata* | *Rolfsiorum* | *P. fructuariae-cellae* | CBS 145110 | Corvina withered grapes stored in  fruit-drying room | Italy | MK039434 | KU554679 | MK045337 | MK520927 |  |
| *Lanata-Divaricata* | *Rolfsiorum* | *P. hainanense* | NN072329 = CBS 144527 | Acidic soil | China | KY495009 | KY495118 | MN969333 | KY495062 |  |
| *Lanata-Divaricata* | *Rolfsiorum* | *P. ochrochloron* | CBS 357.48 | Copper sulphate solution | USA | GU981604 | GU981672 | MN969280 | KF296445 |  |
| *Lanata-Divaricata* | *Rolfsiorum* | *P. piscarium* | CBS 362.48 | Cod-liver oil emulsion | Norway | GU981600 | GU981668 | MN969288 | KF296451 |  |
| *Lanata-Divaricata* | *Rolfsiorum* | *P. pulvillorum* | NRRL 2026 = CBS 280.39 | Soil | UK | AF178517 | GU981670 | MN969289 | KF296452 |  |
| *Lanata-Divaricata* | *Rolfsiorum* | *P. rolfsii* | CBS 368.48 | Pineapple (*Ananas sativus*) | USA | JN617705 | GU981667 | MN969294 | KF296455 |  |
| *Lanata-Divaricata* | *Rolfsiorum* | *P. rotoruae* | CBS 145838 | Pinus radiata timber on ground | New Zealand | MN315103 | MN315104 | MN315102 | MT240842 |  |
| *Lanata-Divaricata* | *Rolfsiorum* | *P. soliforme* | NN072519 = CBS 144482 | Acidic soil | China | KY495038 | KY495147 | MN969337 | KY495047 |  |
| *Lanata-Divaricata* | *Rolfsiorum* | *P. subrubescens* | DTO 188-D6 = CBS 132785 | Soil of *Helianthus tuberosus* field | Finland | KC346350 | KC346327 | KC346330 | KC346306 |  |
| *Lanata-Divaricata* | *Rolfsiorum* | *P. subrutilans* | CGMCC 3.25174  = CS20-14 | Soil | China | OQ870816 | OR051137 | OR051314 | OR051479 |  |
| *Lanata-Divaricata* | *Rolfsiorum* | *P. svalbardense* | CBS 122416 | Glacial ice | Norway | GU981603 | DQ486644 | KC346338 | KF296457 |  |
| *Lanata-Divaricata* | *Rolfsiorum* | *P. terrarumae* | DTO 174-H2  = CBS 131811 | Soil contaminated by heavy  metals | China | MN431397 | KX650295 | MN969323 | MN969185 |  |
| *Lanata-Divaricata* | *Rolfsiorum* | *P. vasconiae* | CBS 339.79 | Acid-washed brown soil | Spain | GU981599 | GU981653 | MN969309 | MN969144 |  |
| *Lanata-Divaricata* | *Rolfsiorum* | *P. tibetense* | CGMCC 3.28597  = XZ5-3 | Rhizosphere soil | China | PQ643284 | PQ519857 | PQ519858 | PQ519859 |  |
| *Lanata-Divaricata* | *Simplicissima* | *P. alagoense* | URM 8086 | Leaf endophyte of *Miconia* | Brazil | MK804502 | MK802333 | MK802336 | MK802338 |  |
| *Lanata-Divaricata* | *Simplicissima* | *P. araracuarense* | CBS 113149 | Forest leaf litter | Colombia | GU981597 | GU981642 | MN969237 | KF296414 |  |
| *Lanata-Divaricata* | *Simplicissima* | *P. brasilianum* | CBS 253.55 | Herbarium specimen | Brazil | GU981577 | GU981629 | MN969239 | KF296420 |  |
| *Lanata-Divaricata* | *Simplicissima* | *P. cataractarum* | KAS 2145  = CBS 140974 | Fallen nuts of C*arya cordiformis* | Canada | KT887847 | KT887808 | KT887769 | MN969180 |  |
| *Lanata-Divaricata* | *Simplicissima* | *P. echinulonalgiovense* | CBS 328.59 | Unrecorded source | Japan | GU981587 | GU981631 | KX961269 | KX961301 |  |
| *Lanata-Divaricata* | *Simplicissima* | *P. fengjieense* | CGMCC 3.25157 = CS15-01 | Soil | China | OQ870765 | OR051156 | OR051333 | OR051489 |  |
| *Lanata-Divaricata* | *Simplicissima* | *P. globosum* | NN072354  = CBS 144639 | Acidic soil | China | KY495014 | KY495123 | KY494954 | KY495067 |  |
| *Lanata-Divaricata* | *Simplicissima* | *P. griseoflavum* | NN072331  = CBS 144525 | Acidic soil | China | KY495011 | KY495120 | MN969331 | KY495064 |  |
| *Lanata-Divaricata* | *Simplicissima* | *P. guangxiense* | NN044175 = CBS 144526 | Soil | China | KY494986 | KY495095 | MN969332 | KY495045 |  |
| *Lanata-Divaricata* | *Simplicissima* | *P. infrabuccalum* | KAS 2181 = CBS 140983 | Carpenter ants (*Camponotus*  *pennsylvanicus*) | Canada | KT887856 | KT887817 | KT887778 | MN969181 |  |
| *Lanata-Divaricata* | *Simplicissima* | *P. jinyunshanicum* | CGMCC 3.25162 = CS02-01 | Soil | China | OQ870766 | OR051157 | OR051334 | OR051490 |  |
| *Lanata-Divaricata* | *Simplicissima* | *P. laevigatum* | NN072364 = CBS 144481 | Acidic soil | China | KY495015 | KY495124 | MN969335 | KY495068 |  |
| *Lanata-Divaricata* | *Simplicissima* | *P. lebretii* | URM 8920 | Cave sediment | Brazil | PP505382 | PP806629 | PP806634 | PP942621 |  |
| *Lanata-Divaricata* | *Simplicissima* | *P. mariae-crucis* | CBS 271.83 | *Secale cereale* | Spain | GU981593 | GU981630 | MN969275 | KF296439 |  |
| *Lanata-Divaricata* | *Simplicissima* | *P. newtonturnerae* | BRIP 74909a | Soil | Australia | OP903478 | OP921964 | OP921962 | OP921963 |  |
| *Lanata-Divaricata* | *Simplicissima* | *P. onobense* | CBS 174.81 | Andosol | Spain | GU981575 | GU981627 | MN969281 | KF296447 |  |
| *Lanata-Divaricata* | *Simplicissima* | *P. panissanguineum* | DAOM250562 = CBS 140989 = KAS 2209 | Soil related to termite mounds | Tanzania | KT887862 | KT887823 | KT887784 | MN969182 |  |
| *Lanata-Divaricata* | *Simplicissima* | *P. paraherquei* | CBS 338.59 | Soil | Japan | AF178511 | KF296465 | MN969285 | KF296449 |  |
| *Lanata-Divaricata* | *Simplicissima* | *P. pedernalense* | F01-11 = CBS 140770 | Whiteleg shrimpheads (*Litopenaeus*  *vannamei*) waste | Ecuador | KU255398 | KU255396 | MN969322 | MN969184 |  |
| *Lanata-Divaricata* | *Simplicissima* | *P. simplicissimum* | CBS 372.48 | Flannel bag | South Africa | GU981588 | GU981632 | MN969297 | JN121507 |  |
| *Lanata-Divaricata* | *Simplicissima* | *P. skrjabinii* | CBS 439.75 | Soil | Russia | GU981576 | GU981626 | MN969299 | EU427252 |  |
| *Lanata-Divaricata* | *Simplicissima* | *P. spinuliferum* | NN072545 = CBS 144483 | Acidic soil with *Litchi chinensis* | China | KY495040 | KY495149 | MN969338 | KY495090 |  |
| *Lanata-Divaricata* | *Simplicissima* | *P. subfuscum* | CN014C9  = CBS 147455 | Soil | South Africa | MT949907 | MT957412 | MT957454 | MT957480 |  |
| *Lanata-Divaricata* | *Simplicissima* | *P. taii* | CGMCC 3.25176 = CS16-09 | Soil | China | OQ870778 | OR051170 | OR051347 | OR051496 |  |
| *Lanata-Divaricata* | *Simplicissima* | *P. tanzanicum* | KAS 1946 = CBS 140968 | Soil related to termite mounds | Tanzania | KT887841 | KT887802 | KT887763 | MN969183 |  |
| *Lanata-Divaricata* | *Simplicissima* | *P. uttarakhandense* | NFCCI 4808 | Soil (garden) | India | MN967315 | MN972443 | MN972445 | MN972447 |  |
| *Lanata-Divaricata* | *Simplicissima* | *P. wotroi* | CBS 118171 | Forest leaf litter | Colombia | GU981591 | GU981637 | MN969313 | KF296460 |  |
| *Lanata-Divaricata* | *Simplicissima* | *P. yuyongnianii* | CGMCC 3.25187  = CS13-01 | Soil | China | OQ870820 | OR051175 | OR051352 | OR051499 |  |
| *Lanata-Divaricata* | *Oxalica* | *P. diatomitis* | CCF 3904  = CBS 140107 | Saline acidic soil | Czech Republic | FJ430748 | HE651133 | LT970912 | LT797560 |  |
| *Lanata-Divaricata* | *Oxalica* | *P. hepuense* | AS3.16039 | Soil of tidal flats | China | MW946994 | MZ004912 | MZ004916 | MW979254 |  |
| *Lanata-Divaricata* | *Oxalica* | *P. jiaozhouwanicum* | AS3.16038 | Soil of tidal flats | China | MW946993 | MZ004911 | MZ004915 | MW979252 |  |
| *Lanata-Divaricata* | *Oxalica* | *P. oxalicum* | NRRL 787 = CBS 219.30 | Soil | USA | AF033438 | KF296462 | MN969283 | JN121456 |  |
| *Lanata-Divaricata* | *Oxalica* | *P. soosanum* | CCF3778 = CBS 140106 | Saline acidic soil | Czech Republic | FJ430745 | FM865811 | LT970913 | LT797561 |  |
| **Aspergilloides* | *Glabra* | *P. glabrum (outgroup)* | CBS 125543 | Unrecorded source | Unknown | GU981567 | GU981619 | KM089152 | JF417447 |  |
