# Supplementary material for: ﻿Two new species of Penicillium (Eurotiales, Aspergillaceae) and the first record of P. danzhouense from mangrove sediment in Thailand, with notes on antibacterial activity
Source: MycoKeys. 2025 Dec 22;126:213–38. doi: 10.3897/mycokeys.126.172211 (PMC12750103; doi:10.3897/mycokeys.126.172211)
Supplement: Supplementary material 2 — Single-gene phylogenies [file mycokeys-126-213-s002.docx]

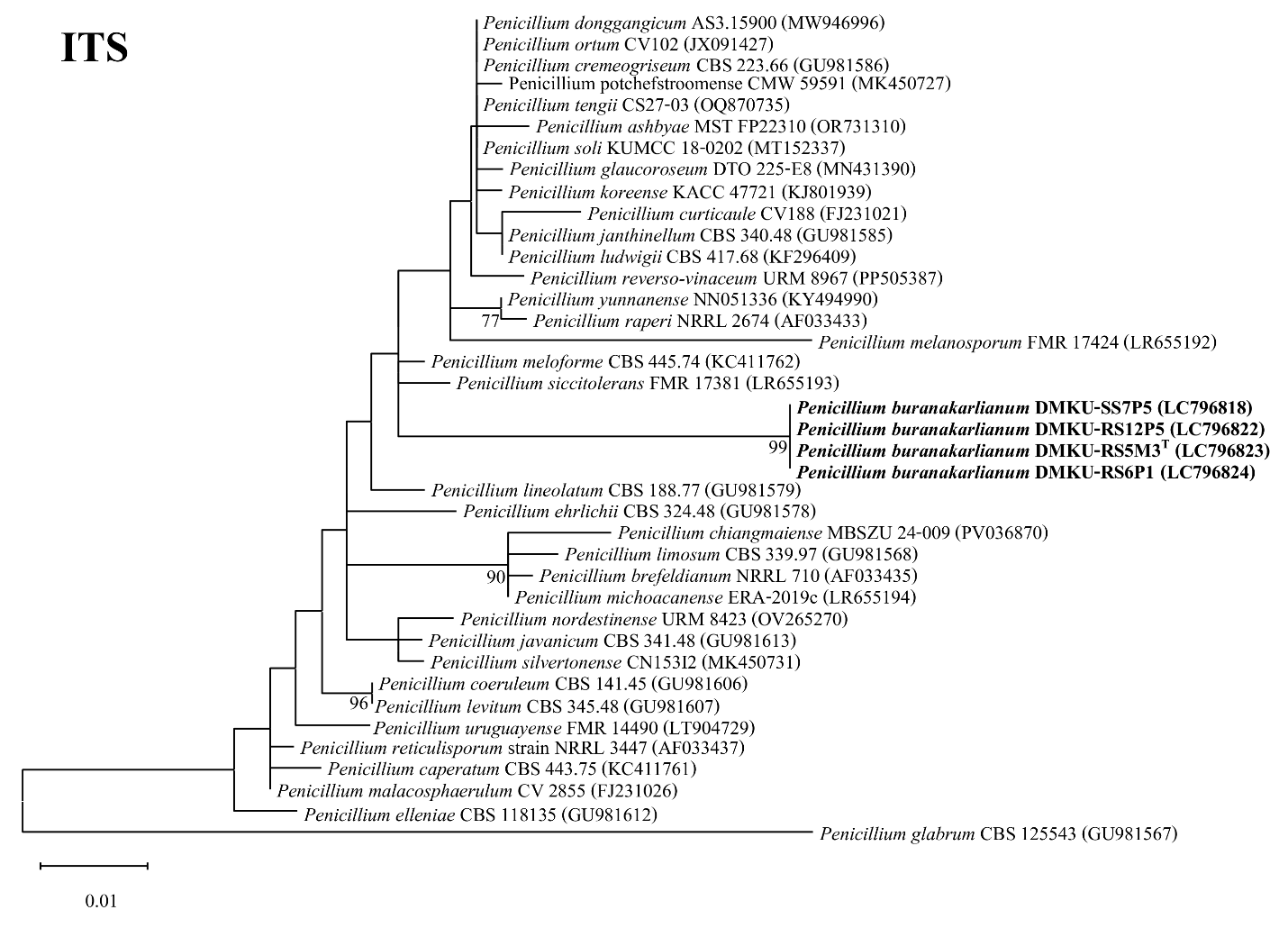


**Figure S1-1.** Maximum likelihood (ML) phylogenetic tree of *Penicillium* species in section *Lanata-Divaricata*, based on ITS. The tree illustrates the phylogenetic positions of the newly described species relative to closely related taxa. Bootstrap support values ≥70% are indicated at the nodes. Newly described species are shown in bold. Strain numbers follow species names, with GenBank accession numbers for ITS provided in parentheses. *Penicillium glabrum* CBS 125543 was used as the outgroup. Scale bar: patristic distance of 0.01.


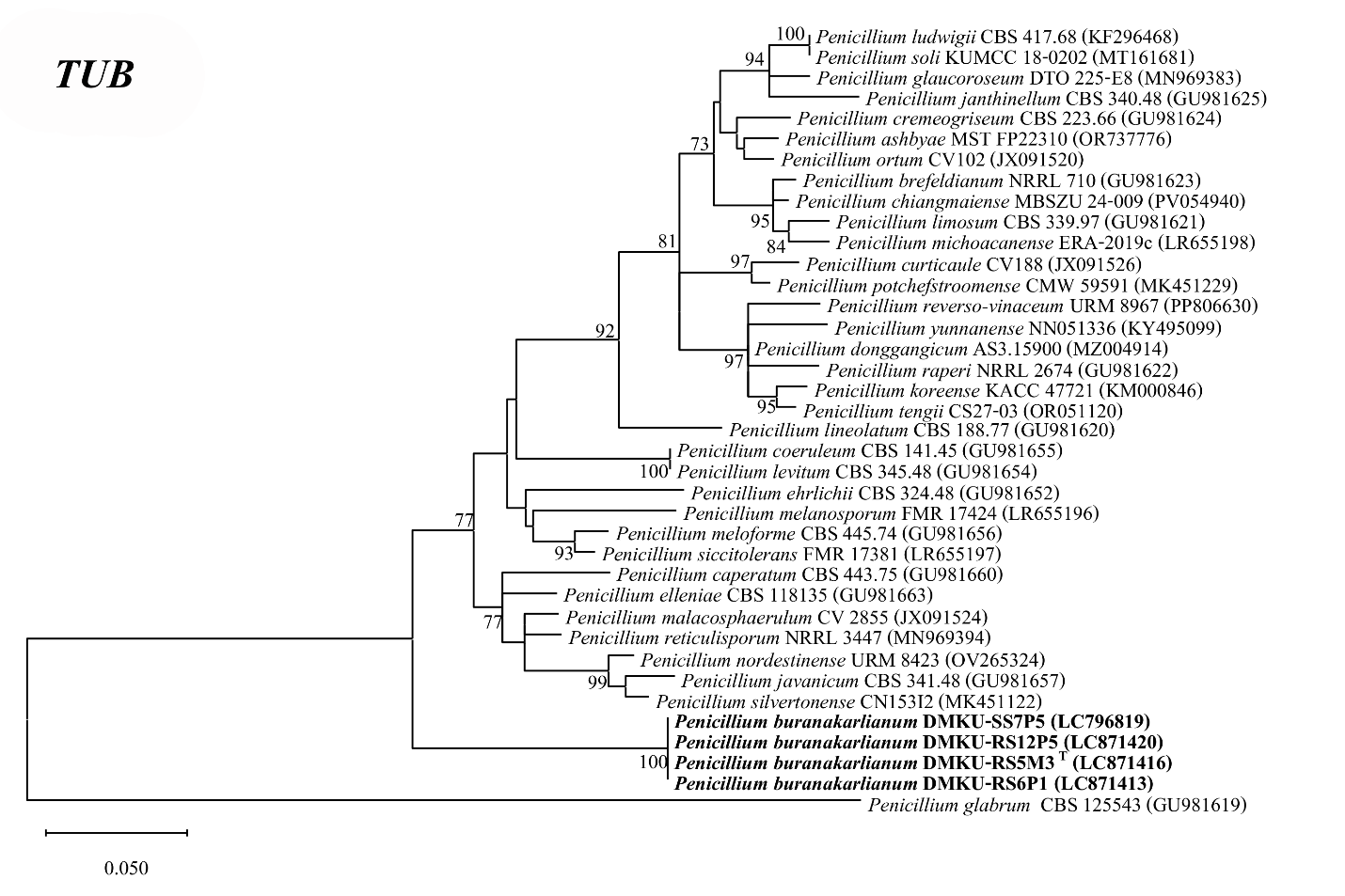


**Figure S1-2.** Maximum likelihood (ML) phylogenetic tree of *Penicillium* species in section *Lanata-Divaricata*, based on *TUB* sequences. The tree illustrates the phylogenetic positions of the newly described species relative to closely related taxa. Bootstrap support values ≥70% are indicated at the nodes. Newly described species are shown in bold. Strain numbers follow species names, with GenBank accession numbers for *TUB* provided in parentheses. *Penicillium glabrum* CBS 125543 was used as the outgroup. Scale bar: patristic distance of 0.050.


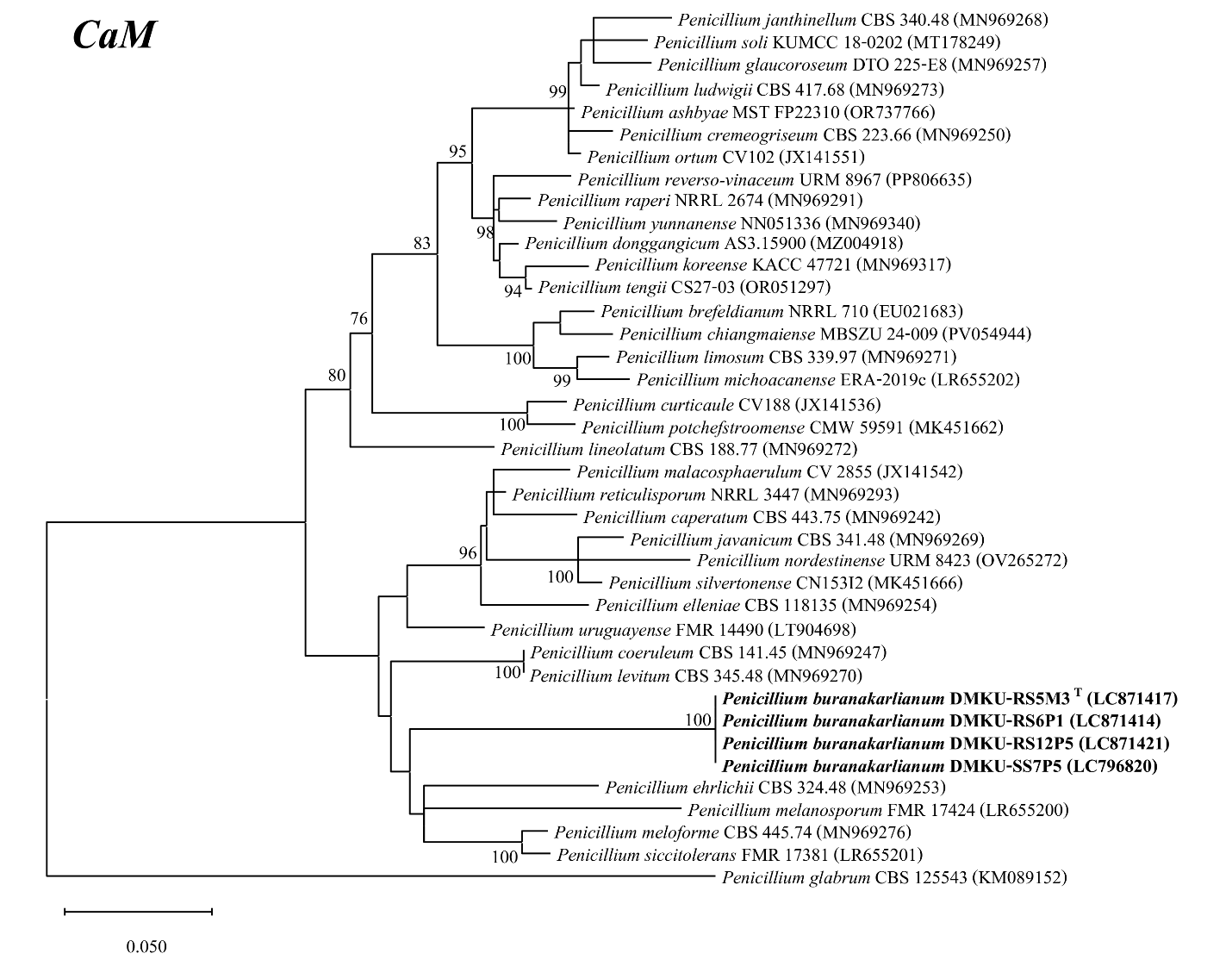


**Figure S1-3.** Maximum likelihood (ML) phylogenetic tree of *Penicillium* species in section *Lanata-Divaricata*, based on *CaM* sequences. The tree illustrates the phylogenetic positions of the newly described species relative to closely related taxa. Bootstrap support values ≥70% are indicated at the nodes. Newly described species are shown in bold. Strain numbers follow species names, with GenBank accession numbers for *CaM* provided in parentheses. *Penicillium glabrum* CBS 125543 was used as the outgroup. Scale bar: patristic distance of 0.050.


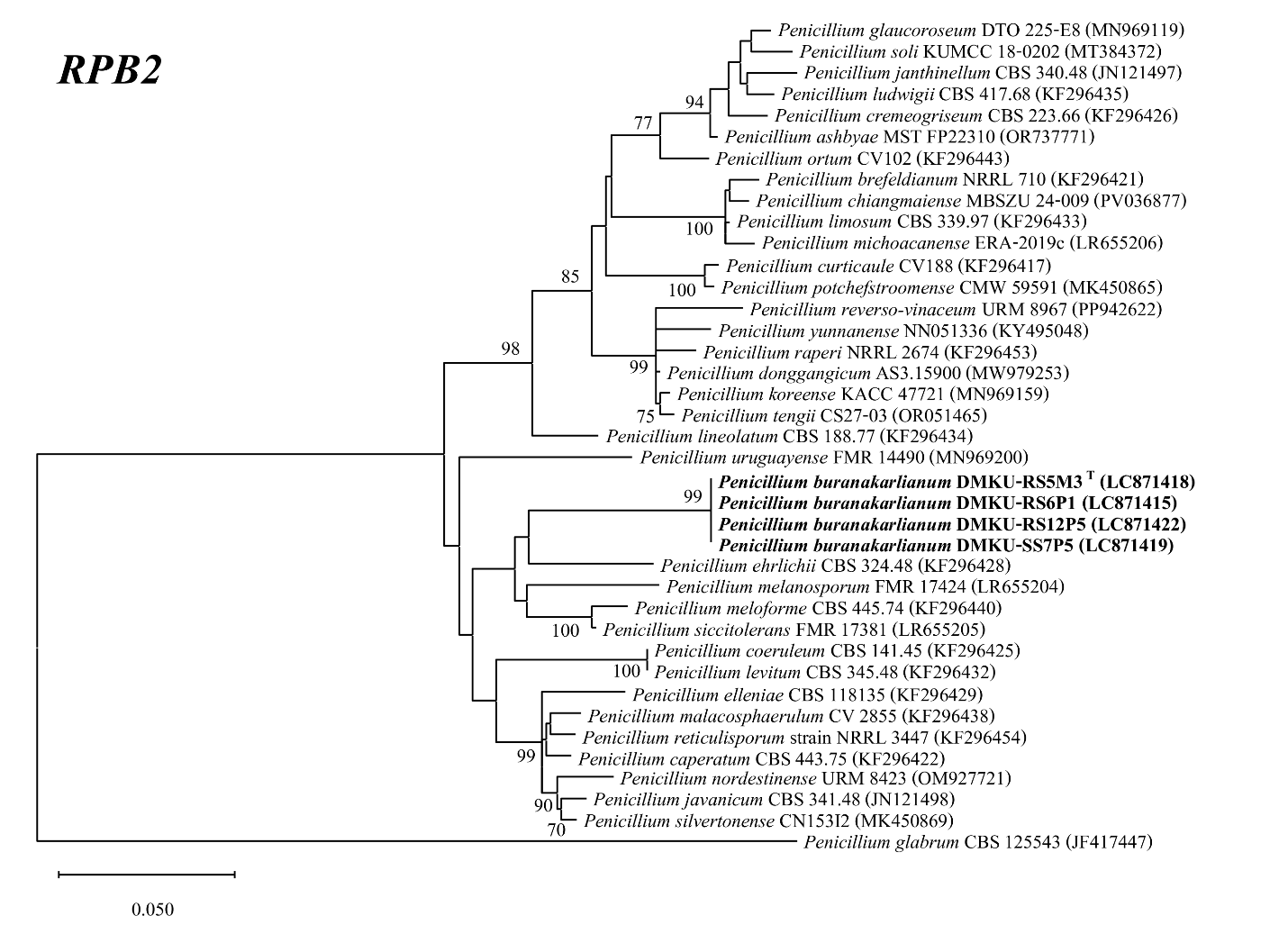
 **Figure S1-4.** Maximum likelihood (ML) phylogenetic tree of *Penicillium* species in section *Lanata-Divaricata*, based on *RPB2* sequences. The tree illustrates the phylogenetic positions of the newly described species relative to closely related taxa. Bootstrap support values ≥70% are indicated at the nodes. Newly described species are shown in bold. Strain numbers follow species names, with GenBank accession numbers for *RPB2* provided in parentheses. *Penicillium glabrum* CBS 125543 was used as the outgroup. Scale bar: patristic distance of 0.050.
